# Monetary incentives increase COVID-19 vaccinations

**DOI:** 10.1126/science.abm0475

**Published:** 2021-10-07

**Authors:** Pol Campos-Mercade, Armando N. Meier, Florian H. Schneider, Stephan Meier, Devin Pope, Erik Wengström

**Affiliations:** 1 Department of Economics, Center for Economic Behavior and Inequality (CEBI), University of Copenhagen, Copenhagen, Denmark.; 2 Unisanté, University of Lausanne, Lausanne, Switzerland.; 3 Faculty of Business and Economics, University of Basel, Basel, Switzerland.; 4 Department of Economics, University of Zurich, Zurich, Switzerland.; 5 Columbia Business School, Columbia University, New York, NY, USA.; 6 Booth School of Business, University of Chicago, Chicago, IL, USA.; 7 National Bureau of Economic Research, Boston, MA, USA.; 8 Department of Economics, Lund University, Lund, Sweden.; 9 Department of Finance and Economics, Hanken School of Economics, Helsinki, Finland.; 10 Knut Wicksell Centre for Financial Studies, Lund University, Lund, Sweden.

## Abstract

The stalling of COVID-19 vaccination rates threatens public health. To increase vaccination rates, governments across the world are considering the use of monetary incentives. Here we present evidence about the effect of guaranteed payments on COVID-19 vaccination uptake. We ran a large preregistered randomized controlled trial (with 8286 participants) in Sweden and linked the data to population-wide administrative vaccination records. We found that modest monetary payments of 24 US dollars (200 Swedish kronor) increased vaccination rates by 4.2 percentage points (*P* = 0.005), from a baseline rate of 71.6%. By contrast, behavioral nudges increased stated intentions to become vaccinated but had only small and not statistically significant impacts on vaccination rates. The results highlight the potential of modest monetary incentives to raise vaccination rates.

## Valuing vaccination

Using money as a motivation for the public to get vaccinated is controversial and has had mixed results in studies, few of which have been randomized trials. To test the effect of money as an incentive to obtain a vaccine, Campos-Mercade *et al*. set up a study in Sweden in 2021, when various age groups were first made eligible to receive the severe acute respiratory coronavirus 2 vaccine (see the Perspective by Jecker). The effect of a small cash reward, around US $24, was compared with the effect of several behavioral nudges. The outcome of this preregistered, randomized clinical trial was that money had the power to increase participation by about 4 percentage points. Nudging and reminding didn’t seem to be deleterious and even had a small positive effect. Of course, the question of whether it is ethical to pay people to be vaccinated like this needs to be addressed. —CA

## Teaser

In Sweden, monetary incentives of US $24 increased vaccination rates from approximately 72% to approximately 76%.

Severe acute respiratory syndrome coronavirus 2 (SARS-CoV-2) and the emergence of new variants are a grave threat to public health. Effective vaccination deployment is essential to mitigate that risk (*1*–*3*). Yet despite widespread awareness and availability of COVID-19 vaccines, many high-income countries struggle to push vaccination rates beyond 70%. At the core of an effective disease containment strategy lie policies that further increase vaccination rates among the hesitant and among people who intend to get vaccinated but do not follow through (*4*–*6*).

Governments and organizations across the world have started using incentives to encourage vaccination, ranging from payments of US$4 (CA$5) in Vancouver and lotteries in Ohio to payments of US$175 (€150) in Greece (*7*, *8*). Many others are now also considering whether to introduce payments for vaccinations. Notably, US President Biden recently urged “…state, territorial, and local governments to provide US$100 payments for every newly vaccinated American, as an extra incentive to boost vaccination rates, protect communities, and save lives” (*9*). Yet, governments and organizations are limited in their ability to properly assess the impact of monetary incentives because they lack control groups that are not exposed to incentives (*10*). Causal evidence examining the effectiveness of introducing payments for COVID-19 vaccinations is lacking.

Here we report findings from a randomized controlled trial (RCT) to study the impact of guaranteed monetary incentives on COVID-19 vaccination. We paid participants, drawn from a general sample of the Swedish population, 200 Swedish kronor (SEK; about US$24) conditional on getting vaccinated. The Swedish setting allows us to link individual-level survey data from the RCT to exhaustive population-wide Swedish administrative records for actual vaccinations collected by public health authorities. We find that the monetary incentives increased vaccination rates by 4.2 percentage points. This is an increase from a 71.6% baseline rate—a rate that is similar to those of other countries in the European Union (EU)—indicating that incentives can increase vaccine uptake even in countries with high vaccination rates.

Our findings are also notable because it is controversial whether monetary incentives to encourage healthier behavior in general, and for COVID-19 vaccination specifically, lead to the desired result. Although monetary incentives have been shown to sometimes encourage healthier behavior (*11*–*15*), incentives can often be ineffective or even counterproductive (*16*–*20*). On the basis of this evidence, many argue that paying people for COVID-19 vaccinations may signal that vaccination is undesirable or even dangerous (*21*, *22*), or that it could crowd out people’s motivation to get vaccinated for the purpose of protecting others (*7*), leading to a decrease in vaccination uptake. By contrast, our results emphasize that modest monetary incentives can increase vaccination rates. However, our findings do not imply that people ought to be paid for getting vaccinated—our paper does not speak to the normative question of whether paying for vaccination is ethically permissible (*23*, *24*).

We also studied the effect of three behavioral nudges on vaccination uptake. Nudges are subtle interventions that do not deny any options or change economic incentives (*25*). They have been used with varying success to alter behaviors (*4*, *26*–*28*). In the context of COVID-19 vaccinations, one study found that in the initial phase of the vaccination rollout, when vaccination rates were around 13%, reminders to book an appointment increased COVID-19 vaccination rates (*29*). However, at the high vaccination rates achieved in many high-income countries, some have argued that nudges may have reached the limit of their potential (*30*). In our trial, we found no statistically significant impact of any of the nudges on vaccination rates.

We conducted the preregistered RCT from May to July 2021, with 8286 participants from 18 to 49 years of age. Participants were recruited from a broadly representative online panel created by Norstat, a large survey company. We sent the survey to each participant as soon as the first Swedish regions opened vaccination for the participant’s age group. In the online survey, we randomized participants into five different treatment conditions and one control condition. Immediately after the treatment, we measured participants’ intentions to get vaccinated against COVID-19. Except for the participants assigned to the no-reminders condition, all participants (even those in the control group) received two reminders to get vaccinated, sent 2 and 4 weeks after taking the survey. In August 2021, the Public Health Agency of Sweden linked the trial data of each participant to the COVID-19 vaccination records collected for all Swedish residents.

Our preregistered main outcome variables are (i) participants’ self-reported intention to get a first dose of a COVID-19 vaccine within 30 days after vaccines become available to them and (ii) whether participants became vaccinated within 30 days, according to the administrative records. All reported results in the text and figures come from ordinary least squares (OLS) regressions with heteroscedasticity-robust standard errors [see supplementary materials (SM) section 1.2.2 for details; all *P* values come from two-sided *t* tests].

In the incentives condition, participants were offered a monetary incentive of SEK 200 (about US$24) if they got vaccinated within 30 days of the vaccine becoming available to them. We used the administrative vaccination records to check uptake.

**Fig. 1.**
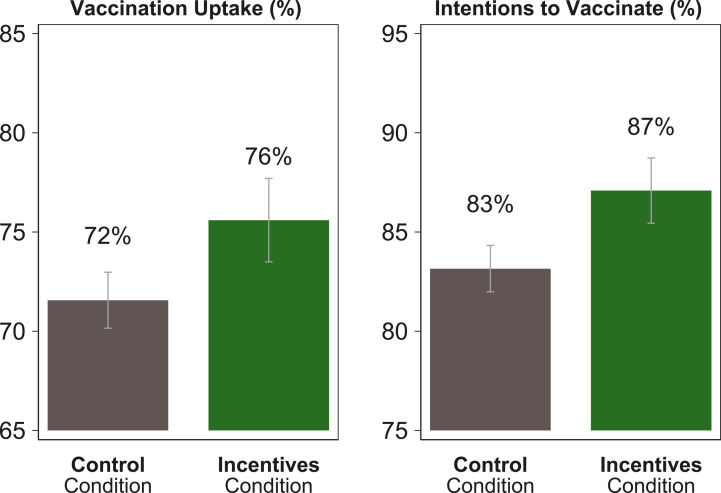
Vaccination uptake and intentions to get vaccinated, among those in the incentives condition and the control condition. The graphs show the proportion of participants in the incentives and control conditions who got vaccinated or intended to get vaccinated, on the basis of survey data from the trial linked to Swedish administrative records on vaccination. “Vaccination Uptake” indicates the proportion of participants who got vaccinated within 30 days of the trial, according to vaccination records. “Intentions to Vaccinate” indicates the proportion of participants who intended to get vaccinated within 30 days of the trial, according to experimental data. Error bars represent normal-based 90% confidence intervals (CIs; mean ± 1.64 SE) from OLS regressions with heteroscedasticity-robust standard errors. *N* = 1131 participants in the incentives condition; *N* = 2778 participants in the control condition.

The incentives condition increased both vaccination intention and actual uptake compared with the control condition (Fig. 1). The proportion of participants who intended to get vaccinated within 30 days was 83.2% in the control condition and 87.1% in the incentives condition, a difference of 3.9 percentage points (*P* = 0.001). The proportion of participants who were vaccinated within 30 days was 71.6% in the control condition and 75.6% in the incentives condition, a difference of 4 percentage points (*P* = 0.009).

**Fig. 2.**
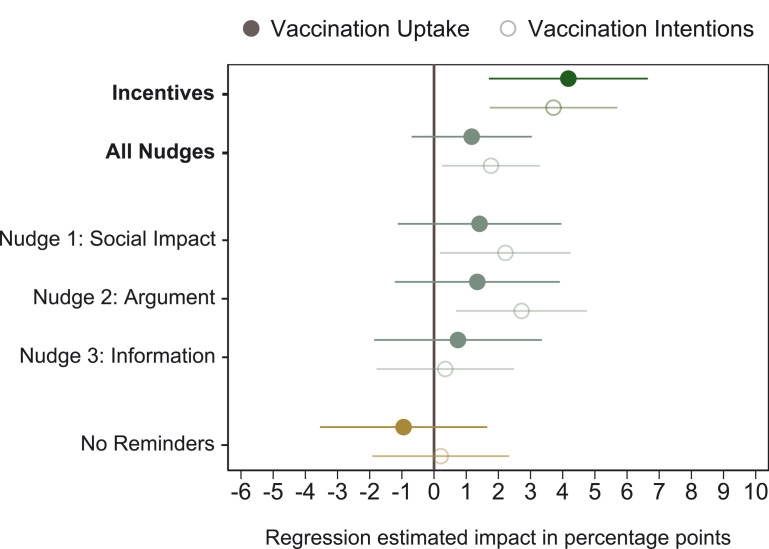
Regression-estimated effects of experimental conditions on vaccination uptake and vaccination intentions versus the control condition. The graph shows regression-estimated effects (OLS regression) of the experimental conditions relative to the control condition, as preregistered. In addition, “All Nudges” denotes the estimate when the social impact, argument, and information conditions are pooled. Filled circles indicate the estimated impact on vaccination uptake within 30 days after participation in the survey (100 if the participant got vaccinated, 0 otherwise). Open circles indicate the estimated impact on intended vaccination uptake (100 if the participant intended to get vaccinated, 0 otherwise) within 30 days. Error bars represent 90% normal-based CIs (coefficient ± 1.64 SE) from OLS regressions with heteroscedasticity-robust standard errors. *N* = 8286 participants.

The effect sizes from our preregistered main specification are shown in Fig. 2. We estimated that receiving monetary incentives for getting vaccinated increased participants’ intentions to become vaccinated by 3.7 percentage points (*P* = 0.002) relative to the control condition. Consistent with these elevated intentions, actual vaccination rates increased by 4.2 percentage points (*P* = 0.005). These results are robust to a battery of robustness checks, such as considering secondary outcome variables, including different sets of control variables, using logistic regressions, correcting for multiple hypothesis testing, and including all participants who went through the experimental intervention but did not finish the survey (SM sections 2.3 and 2.4). We observed similar effects for incentives for vaccination uptake within 10, 20, 30, 40, and 50 days after survey completion (table S7). These results show that monetary incentives not only accelerated immediate vaccination uptake but also increased uptake for at least 50 days.

We collected detailed information on individual characteristics of the participants. We found large baseline differences in vaccination uptake across sociodemographic groups: People with a higher socioeconomic status (college degree, higher income, employed) had higher vaccination rates (SM section 2.6). Notably, and despite the different baseline vaccination rates, we found that monetary incentives boosted vaccination rates similarly across all subgroups (SM section 2.5). This result indicates that monetary incentives have the potential to raise vaccination rates irrespective of people’s background.

We also employed different types of behavioral nudges to persuade participants to become vaccinated (*26*, *31*, *32*): We asked participants (i) to make a list of four people who would benefit from the participant getting vaccinated (social impact condition) (*33*, *34*), (ii) to write down arguments that could best convince another person to get vaccinated (arguments condition) (*27*), and (iii) to participate in a quiz with information on the safety and effectiveness of COVID-19 vaccines (information condition) (*29*). In contrast to the other conditions, a final condition (the no-reminders condition) did not include any nudges or reminders, enabling us to study the impact of reminders on vaccination uptake (*29*).

Some behavioral nudges did statistically significantly increase participants’ intentions to become vaccinated, but none increased actual vaccination uptake (Fig. 2). When we pooled the data from the three nudge conditions (social impact, argument, and information conditions), we found that nudging may elevate vaccination intentions by 1.8 percentage points (*P* = 0.056). However, the increase in intentions translates to only a 1.2 percentage point (*P* = 0.302) rise in vaccination uptake, which is not statistically significantly different from zero. Of the nudges, the social impact and argument conditions had the greatest effect on intentions (social impact: 2.2 percentage points, *P* = 0.072; argument: 2.7 percentage points, *P* = 0.028), but neither of them increased actual vaccination uptake in a statistically significant manner (social impact: 1.4 percentage points, *P* = 0.360; argument: 1.3 percentage points, *P* = 0.388). The comparison of the no-reminders condition with the control condition indicated that reminders did not substantially affect vaccination rates (*P* = 0.594). Moreover, there is no statistically significant difference between the no-reminders condition and the three nudge conditions (*P* = 0.243). We did not find any statistically significant or economically meaningful differences across sociodemographic groups, such as those categorized by immigration status, income, or gender (table S21).

Hence, we found that monetary incentives had greater effects on vaccination uptake than did behavioral nudges. Although the preregistered main analysis focused on the comparison between each of the experimental conditions and the control condition, we were also able to study the impact of the incentives condition relative to the three nudges. We found that the incentives condition had a larger impact on vaccination uptake than the three nudges pooled (difference of 3.1 percentage points, *P* = 0.038).

**Fig. 3.**
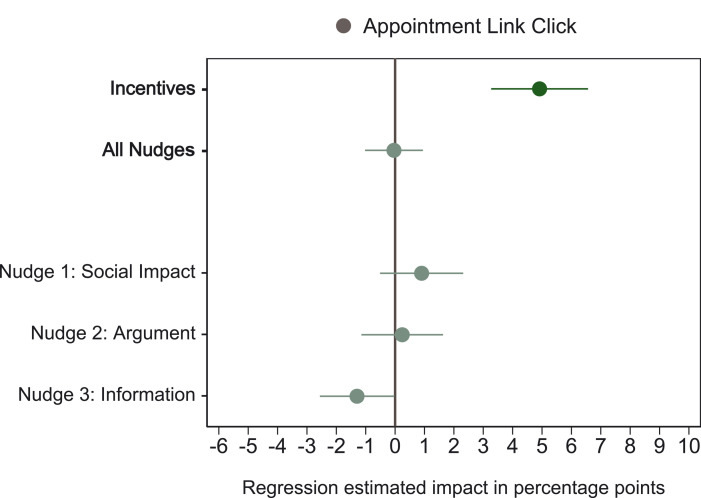
Regression-estimated effects of experimental conditions on whether participants clicked a link to a website with information for scheduling a vaccine appointment. The graph shows regression-estimated effects (OLS regression) of the experimental conditions relative to the control condition, as preregistered. In addition, “All Nudges” denotes the estimate when the social impact, argument, and information conditions are pooled. Circles indicate the estimated impact of each experimental condition on the probability of clicking the appointment link (100 if the participant clicked the link, 0 otherwise). The no-reminders condition is not included because this condition did not include the link. Error bars represent 90% normal-based CIs (coefficient ± 1.64 SE) from OLS regressions with heteroscedasticity-robust standard errors. *N* = 7288 participants.

We also found a difference between monetary incentives and behavioral nudges in terms of whether, at the end of the survey, participants clicked a link to a website with information to schedule a vaccine appointment (Fig. 3). In the incentives condition, participants were more likely (by 4.9 percentage points, *P* < 0.001) to click on the link, whereas participants in the nudge conditions did not click on the link more often than those in the control condition (−0.08 percentage points, *P* = 0.889). Thus, participants were more likely to click the appointments link in the incentives condition than in the three behavioral nudge conditions (4.8 percentage points, *P* < 0.001).

In sum, our study reveals that even modest monetary incentives can boost COVID-19 vaccination rates. We found that payments of SEK 200 (≈US$24) raised COVID-19 vaccination rates by 4 percentage points. Our trial shows that incentives can increase vaccination uptake even when baseline vaccination rates are high. By contrast, behavioral nudges had small and not statistically significant effects on vaccination rates.

A natural question is whether paying people to get vaccinated is cost-effective for governments. In addition to the direct benefits of saving lives, boosting vaccination rates leads to indirect benefits such as enhanced population immunity, lower hospitalization rates and medical costs, and economic growth. It is beyond the scope of this report to provide a comprehensive analysis of cost-effectiveness, but SM section 2.9 offers some perspectives on why the intervention likely is cost-effective. A key consideration is that paying for vaccination carries much lower costs for society than the sum of all payments—because money is transferred from the government to the citizens, the money paid is not lost.

Our study has several limitations. First, we tested only one size of monetary incentive. Companies and governments around the world have proposed incentives that range from less than US$1 in Philadelphia and US$29 (€25) in Serbia to US$100 in New York. Our trial cannot shed light on whether smaller or larger incentives would be more effective. We also cannot assess the effectiveness of other ways of incentivizing people, such as raising health insurance premiums for the unvaccinated. Second, during summer 2021 Sweden had a vaccination rate in line with the EU average, but countries differ greatly in the proportion of vaccinated population, and the effect of incentives may vary depending on vaccination rates. Relatedly, we offered incentives when the vaccine rollout was starting; results may differ if monetary incentives are offered later—for example, because the reluctance of unvaccinated people may grow over time. Third, the existence of monetary incentives could potentially crowd out people’s willingness to get vaccinated in the future (e.g., booster shots) without getting paid. Finally, people might react differently depending on who provides monetary incentives and the corresponding level of trust in receiving the promised payments. In our case, researchers provided incentives, but the effects may differ if incentives are offered by governments or companies.

Despite these limitations, our preregistered trial yields a clear result: Guaranteed incentives can increase COVID-19 vaccination rates. As the COVID-19 pandemic continues, incentives could be an effective tool to reduce COVID-19 spread and fatalities.

## Supplementary Materials

### This PDF file includes:

Materials and Methods Supplementary Text Figs. S1 to S12 Tables S1 to S28 References (*37*–*44*)

### Other Supplementary Material for this manuscript includes the following:

MDAR Reproducibility Checklist
