# Supplementary material for: Monetary incentives increase COVID-19 vaccinations
Source: Science. 2021 Oct 7;374(6569):879–82. doi: 10.1126/science.abm0475 (PMC10765478; doi:10.1126/science.abm0475)
Supplement: Supplementary file 1 — Materials and Methods Supplementary Text Figs. S1 to S12 Tables S1 to S28 References (37–44) [file science.abm0475_sm.pdf]

Supplementary Materials for

Monetary incentives increase COVID-19 vaccinations

Pol Campos-Mercade *et al.*

Corresponding authors: Pol Campos-Mercade, pcm@econ.ku.dk; Armando N. Meier, armando.meier@unil.ch; Florian H. Schneider, florian.schneider2@uzh.ch; Stephan Meier, sm3087@gsb.columbia.edu; Devin Pope, devin.pope@chicagobooth.edu; Erik Wengström, erik.wengstrom@nek.lu.se

Science **374**, 879 (2021)
DOI: 10.1126/science.abm0475

The PDF file includes:

Materials and Methods
Supplementary Text
Figs. S1 to S12
Tables S1 to S28
References

Other Supplementary Material for this manuscript includes the following:

MDAR Reproducibility Checklist

Detailed list of contents:

1 Materials and Methods **6**

1.1 Ethics approval 6

1.2 Details on the RCT 6

1.2.1 Overview 6

1.2.2 Data analysis and pre-registration 8

1.2.3 Survey and exclusion criteria 9

1.2.4 Online survey 11

1.2.5 Experimental conditions and measurement of intentions 11

1.3 Swedish administrative records for vaccinations and measurement of vaccination uptake at the indidvidual level 14

2 Supplementary Text **15**

2.1 Details on data analysis and pre-analysis plan 15

2.1.1 Further details on sample, outcomes, and control variables 15

2.1.2 Summary of preregistered hypotheses and specification 19

2.1.3 Details on the Swedish vaccination rollout and timing of the experiment . . 22

2.2 Summary statistics, balance, and sample 25

2.2.1 Summary statistics 25

2.2.2 Balance 28

2.2.3 General population sample 29

2.3 Main results and robustness 30

2.3.1 Main outcome variables 31

2.3.2 Secondary outcome variables 34

2.3.3 Considering different time windows 37

2.3.4 Alternative vaccination uptake definitions 39

2.3.5 Results comparing incentives to all nudges 41

2.4 Further robustness checks 42

2.4.1 Accounting for rollout dynamics and different sets of controls 42

2.4.2 Logit regressions 48

2.4.3 Sample weights 49

2.4.4 Different inclusion criteria 50

2.4.5 Multiple hypothesis testing for vaccination uptake 54
2.5 Treatment effects based on participants' characteristics 55
2.5.1 Heterogeneities based on sociodemographics 57
2.5.2 Heterogeneities based on economic preferences, personality traits, and COVID-19 related variables 59
2.6 Predictors of vaccination uptake and the intention-behavior gap 60
2.6.1 Predictors of vaccination uptake 60
2.6.2 Predictors of the intention-behavior gap 65
2.7 Survey responses to the nudge conditions 67
2.7.1 Social impact condition 67
2.7.2 Argument condition 67
2.7.3 Information condition 67
2.8 Expert predictions of treatment condition impacts on vaccination uptake and intentions vs. actual impacts 70
2.9 Perspectives on cost-effectiveness 72
2.10 Experimental instructions 74

Figs. S1 to S12

S1 Timeline, exclusion criteria and randomization of RCT. 7
S2 Distribution of the days between trial participation and vaccination rollout in a participants' region 24
S3 Distribution of observed days between trial participation and linkage with administrative records 24
S4 Vaccination uptake by experimental condition 32
S5 Vaccination intentions by experimental condition 33
S6 Clicks on the appointment link by experimental condition 36
S7 Proportion of participants who got vaccinated per day after the trial 38
S8 Treatment effects of incentives across sociodemographic groups 57
S9 Beliefs about COVID-19 vaccine effectiveness in preventing deaths, correct answer: 91-100% 68
S10 Beliefs about the number of deaths caused by COVID-19 vaccines (Pfizer), correct answer: 0 69

S11 Actual vs. predicted impact on vaccination uptake 71
S12 Actual vs. predicted impact on intentions 71

Tables S1 to S28

S1 Table on vaccination rollout dates 23
S2 Summary Statistics 25
S3 Balance checks 28
S4 RCT sample and the Swedish population 29
S5 Treatment effects on vaccination uptake and intentions 31
S6 Treatment effects using the secondary outcome variables 35
S7 Treatment effects on vaccination uptake by different time windows 37
S8 Treatment effects on vaccination uptake, different definitions 40
S9 Comparing incentives to nudges 41
S10 Treatment effects controlling for each specific age interacted with region 43
S11 Treatment effects on incentives and all nudges controlling for the timing of the survey 44
S12 Treatment effects on all conditions controlling for the timing of the survey 45
S13 Treatment effects on incentives and all nudges including different sets of controls . 46
S14 Treatment effects on all conditions including different sets of controls 47
S15 Treatment effects using logit regressions 48
S16 Treatment effects with sample weights 49
S17 Treatment effects including participants who did not finish the survey 50
S18 Treatment effects including participants who were already vaccinated 51
S19 Treatment effects including participants who participated more than once 52
S20 Treatment effects including participants who could not be linked 53
S21 Heterogeneous treatment effects based on sociodemographics 58
S22 Heterogeneous treatment effects based econ. pref, personality, and COVID-19 variables 59
S23 Predictors of vaccination uptake: economic preferences 61
S24 Predictors of vaccination uptake: COVID-19 vaccination beliefs 62
S25 Predictors of vaccination uptake: sociodemographics 63
S26 Predictors of intention-behavior gap: economic preferences 64
S27 Predictors of intention-behavior gap: COVID-19 related variables 65

S28 Predictors of intention-behavior gap: sociodemographics 66

1 Materials and Methods

1.1 Ethics approval

The Swedish ethical review authority (Etikprövningsmyndigheten) approved the protocols of our randomized controlled trial (reference number 2021-01658). Informed consent was obtained from all study participants as part of the enrollment process. People for whom the Public Health Agency of Sweden (Folkhälsomyndigheten) recommended against vaccinating at the time of the trial (because of potentially heightened health risks) were prevented from participating in our study (see below for details).

1.2 Details on the RCT

1.2.1 Overview

We conducted a pre-registered RCT with a general population sample of Swedish residents, see Figure S1 for the trial structure. In an online survey conducted in collaboration with the survey companies Enkätfabriken and Norstat, we measured participants' sociodemographics, economic preferences, personality traits, and knowledge and worries related to COVID-19 vaccines. We then randomly allocated them to interventions designed with the goal to increase vaccination uptake. Finally, we measured participants' intentions to get vaccinated against COVID-19. We match the data from the online survey with population-wide Swedish administrative records for vaccinations, which allow us to examine whether the participants got vaccinated.

Figure S1: Timeline, exclusion criteria and randomization of RCT.

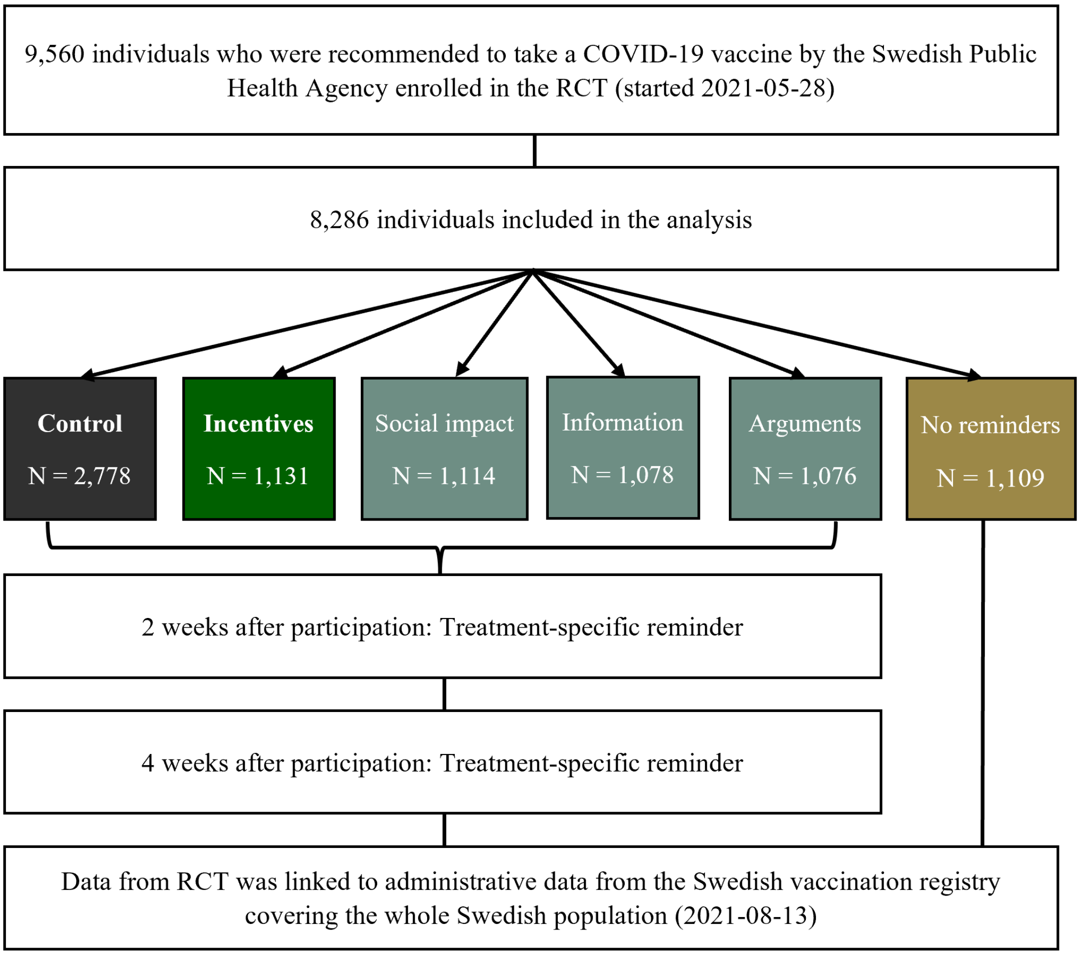

Note: This figure shows the timeline, eligibility criteria for participation, the number of participants excluded from the analysis, and the number of participants in each experimental condition. The respondents for the trial were recruited from a general population sample of Swedish residents aged 18 to 49. The trial started on May 28 and concluded on July 13, 2021. Individual linkage with population-wide administrative records on vaccinations was done by the Swedish Public Health Agency on August 13. Participants were excluded from the analysis due to i) not completing the online survey (N=502), ii) the reported social security number could not be matched with the vaccination registry (N=229), ii) already receiving a first dose before completing the survey (N=224), and iv) participated multiple times in the RCT and got assigned to different behavioral interventions (N=319). In SM Section 2.4.4, we show that including excluded participants does not affect our results. We oversampled the control condition to increase power, see the discussion in Section 1.2.4. Participants in the incentives condition received a guaranteed monetary payment of SEK 200 ($24) for getting vaccinated within 30 days. The social impact condition highlighted the positive social impact of the vaccine by making participants write a list of 4 people who could benefit from the participant getting vaccinated. The information condition consisted of a quiz, providing information about COVID-19 vaccine safety and effectiveness. In the arguments condition, participants had to write down arguments that could best convince another person to get the COVID-19 vaccine as soon as possible. The no-reminders condition did not encourage participants to vaccinate, did not include a link with information on where to vaccinate, and did not use two reminders which were used in all other conditions.

1.2.2 Data analysis and pre-registration

We pre-registered the data collection and analysis at the AEA RCT Registry (`https://doi.org/10.1257/rct.7652-2.0`). We provide a detailed discussion of the pre-analysis plan and the data analysis in SM Section 2.1. The purpose of our RCT was to study the impact of monetary incentives and different behavioral nudges to increase COVID-19 vaccination uptake. All code and data for reproducing tables and figures in the manuscript and the supplementary materials can be accessed through the following link: `https://doi.org/10.5281/zenodo.5529625` (*35*).

To estimate treatment effects, we pre-registered using ordinary least squares (OLS) regressions with heteroscedasticity-robust standard errors, controlling for gender, age, region, interactions between age and region, being in an at-risk group for COVID-19, civil status, having children in the household, employment status, education, parents' place of birth, and income (see SM Section 2.1.1 for definition of all variables). We present results with the exact set of pre-registered controls in the main text and in SM Section 2.3. In SM Section 2.4 we show that results are robust to including different sets of control variables, using logit regressions, correcting for multiple hypothesis testing, using different sample weights, and using different inclusion criteria. All analyses show consistent and statistically significant impacts of the incentives condition.

As secondary analyses, we also pre-registered that we would study whether interventions differentially impacted participants based on their economic preferences, personality traits and sociodemographics, and whether such preferences, traits and sociodemographics predict vaccination uptake. We provide these analyses in SM Sections 2.5 and 2.6. In addition, we further provide the same analyses also for sociodemographics, COVID-19 disease history, COVID-19 risk-group status, and vaccine attitudes.

We pre-registered self-reported vaccination intentions (Vaccination intention) and actual vaccination uptake (Vaccination uptake) as the two main outcome variables.

Vaccination intention corresponds to participants' answer in the survey question "Do you think you will get a first shot of a COVID-19 vaccine within the first 30 days after a vaccine becomes available to you?", where the response was binary.

Vaccination uptake corresponds to whether participants got vaccinated within 30 days after they filled out the survey. In the trial, we encouraged participants to get a COVID-19 vaccine as soon as possible, ideally within the first 30 days after it became available to them. In line with the design, we pre-registered that we would examine whether participants got vaccinated within

30 days after the vaccine became available. However, the rollout turned out to be much more complex than we expected (see SM Section 2.1.3).[1] Although we were able to recover many of the specifics of the regional vaccination rollout through news feeds from the regional public health authorities and local newspapers, our measure of when vaccines became available for each participant is imperfect. Hence, before linking the trial data to the vaccination records, we decided that we would slightly deviate from the pre-analysis plan and instead focus in the main analysis on whether each participant got vaccinated within 30 days after survey completion, with the advantage of having an exact and easily interpretable measure. SM Section 2.3.2 presents results for the measure on vaccination uptake within 30 days of availability, relying on the information on the rollout that we were able to recover (see SM Section 2.1.3). Importantly, across both ways of specifying vaccination uptake, the results are equivalent in terms of statistical significance and estimated impact.

1.2.3 Survey and exclusion criteria

The participants were recruited from a general population panel in Sweden by the survey company Norstat. Norstat actively recruits people via phone calls to create a representative panel in terms of age, region, and gender. In our case, we asked the company to recruit as many participants between 18-49 years old as possible (this request lead to a slight over-representation of female participants, as reported in Section 2.2.3). Participants were asked to fill out an online survey, and responses were collected between May 28, 2021 and July 13, 2021. The Swedish Public Health Authority then linked the trial data at the individual-level to vaccination uptake as indicated by Swedish administrative records on August 13. We excluded participants who were not recommended to take a COVID-19 vaccine by the Public Health Agency of Sweden at the time of the trial. To do so, we asked participants whether they were already vaccinated, were pregnant, had previously experienced an allergic reaction that required hospital care, or had ever experienced a severe allergic reaction after they got a vaccine. Those who answered affirmatively in one of these questions were excluded from the survey.

[1] At the time we wrote the PAP in May 2021, the region- and age-specific vaccination strategy of Sweden was not yet defined. Hence, we discussed in the PAP that we would need to adapt parts of the analysis to adequately fit Sweden's vaccination rollout. It turned out that each region used very different age-specific rollout strategies, with some using 10-year age groups, others 5-year age groups, and others alternating and even opening vaccination appointments moving from one specific age to the next within a matter of days. Additionally, most regions allowed Swedish residents from other regions to vaccinate in their region, further complicating the definition of when a vaccine is available to each participant.

Sweden's vaccination strategy differed across regions. Regions chose similar strategies, starting with vaccinating the elderly and then gradually lowering the age threshold (see SM Section 2.1.3 for more details). We wanted to reach participants around the time when vaccines became available to them. We therefore fielded the survey in three age-group-specific waves in accordance with the Swedish vaccination rollout. For each age group, we gathered data over the course of two weeks. We collected the first wave of data from May 28 to June 11 and only invited participants between the ages of 40 and 49. Then, we collected the second wave of data from June 17 to July 1 and only invited participants between the ages of 30 and 39. Last, we collected the third wave of data from July 1 to July 13 and only invited participants between the ages of 18 and 29.[2] We did not recruit participants older than 50 because the rollout for ages 50 and older started before we received ethics approval. In our analysis, we account for the age-specific vaccination strategy of Sweden and the timing of the survey waves by controlling for age-specific effects (age fixed effects), region-specific effects (region fixed effects), and the interaction of the two (region x age fixed effects), as pre-registered in our pre-analysis plan. The results are robust to a variety of different specifications including further accounting for the dynamics of the vaccination rollout, see SM Section 2.4.1.

We obtained responses from 9,560 individuals to the online survey of which we excluded 502 from the analysis that did not complete the online survey (N=502). In SM Section 2.4.4, we show that including all participants who went through the experimental intervention but did not finish the rest of the questionnaire does not affect our results. We also exclude 229 participants who completed the survey but whose reported social security number did not match the vaccination registries (likely because of typos in the reported social security number). We exclude 319 participants who answered the survey more than once, but were exposed to different treatment conditions. Last, we exclude the 224 observations from participants who according to the administrative records had already received a first shot before completing the survey. In SM Section 2.4.4, we show that our results are equivalent when using different sample specifications. Our main analysis sample includes 8,286 participants.

Descriptive statistics of the sample are presented in SM Section 2.2.1. In comparison with the Swedish population, our sample is representative with respect to age, income and region. However, we have a slight overrepresentation of women and people with a college education, and an

[2]Our data-analysis confirms that we did indeed launch the survey waves around the time when most regions opened vaccination for the respective age groups. Using our constructed measure of when vaccination was available for each region and age group, we find that participants answered the survey on average 1 day before the rollout started for their age and region. We find that 90% of the sample answered the survey +/- 15 days from when the rollout started for them.

underrepresentation of people with immigrant background (see SM Section 2.2.3). In SM Section 2.4.3, we show that results do not change when using sampling weights to adjust for the misrepresentation. In addition, we find that participants' sociodemographics were comparable across experimental conditions (see SM Section 2.2.2).

1.2.4 Online survey

We first asked all participants for their social security number, which we used to match their survey responses to their administrative records on COVID-19 vaccination. Next, participants answered a series of survey questions on economic preferences and personality traits. We measured altruism, reciprocity, trust, patience, and risk-affinity using experimentally validated measures from the Global Preference Survey (*37*). We also measured a proxy for the tendency to procrastinate (time-inconsistency) taken from the psychology literature (*38*). Finally, we collected self-reported adherence to social norms using a question from the Schwartz Value Survey, which has been extensively used in the World Values Surveys. We proceeded by collecting participants' safety perception of COVID-19 vaccines, knowledge about vaccines in general and worries about side effects of COVID-19 vaccines, fear of needles, history of COVID-19 infection, vaccine eligibility, risk group status, and sociodemographics. We give the complete questionnaire translated to English, including all questions and all interventions, in SM Section 2.10.

1.2.5 Experimental conditions and measurement of intentions

After the first part of the survey, we randomly assigned participants to one of six experimental conditions. We allocated 1/3 of all participants to the *control condition*. We oversampled the control condition as pre-registered because we were particularly interested in understanding which experimental conditions would increase vaccination uptake relative to the *control condition*. Hence, the main specification compares the experimental conditions to the *control condition*. Given our interest in this comparison, power calculations indicated that oversampling of the *control condition* increased power. As in all experimental conditions except the *no-reminders condition*, we encouraged participants to vaccinate within 30 days after the vaccine becomes available to them ("We would like to encourage you to get a COVID-19 vaccine as soon as possible, ideally within the first 30 days after the vaccine becomes available to you"), we included a link where they can book an appointment for their vaccination, and we sent two reminders with the encouragement and the link within the following four weeks.

To study the impact of monetary incentives on vaccination uptake, we allocated 2/15 of participants to the *incentives condition*. In the *incentives condition*, we offered participants SEK 200 ($\approx$ USD 24) if they got vaccinated within 30 days after they were eligible to get vaccinated or, in case they were already eligible, 30 days after they filled out the survey. More concretely, we wrote: "We offer you SEK 200 if you get a first shot of a COVID-19 vaccine within the first 30 days after the vaccination becomes available to you." We informed participants that we would check whether they got vaccinated using administrative data. We further included a small text box in the survey where one of the authors, Erik Wengström, signed that he guaranteed that the participant would receive the payment if he/she vaccinated within the time frame. We made the payments with a voucher called SuperPresentKort, which can be universally used in large retail stores and restaurant chains in Sweden, essentially working like cash.

To study the impact of different behavioral nudges on vaccination uptake, we allocated 2/15 of participants to each of the following three experimental conditions.

In the *social impact condition*, we asked participants to make a list of 4 people that would benefit if the participant would get vaccinated. The purpose of this intervention was to make participants aware of the social impact of getting vaccinated. We also provided participants with an illustration of such a list, written by one of the authors of this study, which likely reinforced the perceived social impact of vaccination. This intervention was motivated by prior evidence showing that prosocial motives can play an important role for flu and polio vaccination uptake (*32, 33, 39*) as well as for health behaviors in the context of the COVID-19 pandemic (*34*).

In the *arguments condition*, we asked participants to write down an argument that could convince another person to get vaccinated as soon as possible. We also gave participants the opportunity to share this argument with a person who did not plan to get vaccinated, which 80% of participants did. We then selected the arguments of ten participants and, if the participants agreed, shared the argument with a person on Amazon Mechanial Turk. The idea behind this intervention is that the process of coming up with an argument and sharing it might convince the participants themselves of the importance of getting vaccinated (*27*).

In the *information condition*, we used a quiz to inform people about the safety and effectiveness of the COVID-19 vaccines with the goal of increasing vaccination uptake by correcting misconceptions. We informed participants that the provided information was confirmed as accurate by Prof. Niklas Arnberg, a respected virologist in Sweden based at the University of Umeå. First, we asked participants how effective the COVID-19 vaccines offered in Sweden for their age group (Pfizer-

BioNTech and Moderna) are in reducing deaths from COVID-19. We then gave them a correct answer, based on the evidence available at the time of the trial, which was that the Pfizer-BioNTech vaccine prevented 97% of deaths from COVID-19 (*40*). Second, we asked participants how many deaths had occurred because of side effects from a Pfizer-BioNTech or Moderna COVID-19 vaccine. Again, we provided the correct answer based on the evidence available at the time of the trial, which was that there were no confirmed cases of deaths due to the Pfizer-BioNTech or Moderna COVID-19 vaccines neither in the US nor in the EU. Our data confirms that people indeed underestimated vaccine safety and effectiveness, over 80% chose a wrong answer in either question, hence the condition could potentially shift peoples' perceptions and impact vaccination uptake (see SM Section 2.7.3). This condition is motivated by the often-discussed need of correct information to fight misinformation (*29, 41*).

Finally, we allocated 2/15 of participants to the *no-reminders condition*. In contrast to all other conditions, the *no-reminders condition* does not include an encouragement to vaccinate, does not include a link to schedule an appointment, and does not include reminders. The comparison of this condition with the control condition therefore allows us to assess the impact of these factors. This experimental condition is motivated by Dai and coauthors (*29*), who find that reminders can boost COVID-19 vaccination uptake.

One potential concern is that the different conditions may imply different intellectual effort from the participants. For instance, the three nudge conditions (social impact, argument, and information conditions) could require more effort from the participants than the incentives condition. This could be problematic if for example participants in the nudges conditions were less likely to finish the survey due to such effort. However, the data show that participants assigned to the control group, any of the nudges, and the incentive conditions finished the survey at very similar rates (96%-98%), suggesting no substantial differences in the effort needed to fill out the survey. Moreover, including participants who did not finish the survey does not change the estimates (SM Section 2.4.4).

After the intervention, we asked all participants about their intentions to get vaccinated. For the main pre-registered intention measure, participants were asked whether they think that they will get a first shot of a COVID-19 vaccine within the first month after the vaccine becomes available to them. We also collected two secondary intention measures: we asked participants about i) the chance that they will get a first shot of a COVID-19 vaccine within the first month after the vaccine becomes available to them and ii) when they think they want to get a first shot of a COVID-19

vaccine. In the main text we focus on the main outcome measure, but the findings are similar for the two secondary measures (SM Section 2.3.2).

At the end of the survey, we provided participants (except those in the *no-reminders condition*) with a link to a website of their regional health authorities where they could receive information about how to sign up for a vaccination appointment. We recorded whether participants clicked on the link, which we pre-registered as a secondary outcome variable.

We sent participants (except in the *no-reminders condition*) two treatment-specific reminders, the first around two weeks and the second around four weeks after participation in the online survey. In the *control condition*, the reminder encourages participants to get vaccinated as soon as possible, ideally within 30 days. In the *incentive*, *social impact*, *argument* and *information condition*, we added a treatment-specific sentence, reminding participants of the incentives, social impact, vaccine safety and effectiveness, and the argument, respectively. See SM Section 2.10 for the exact wording of all reminders.

1.3 Swedish administrative records for vaccinations and measurement of vaccination uptake at the indidvidual level

We use administrative data from national vaccination registers comprising all residents of Sweden. As it is not possible to opt out of or delete records in the vaccination registry, the administrative records include the date of each vaccination of each Swedish resident. In the administrative data, we see whether and when each participant got vaccinated. The Public Health Agency of Sweden linked our trial data at the individual-level with the administrative data on August 13. As the trial ended on July 13, we observe for participants whether and when they got a first COVID-19 vaccine shot within a time window of 30 days after participation in the trial.

2 Supplementary Text

2.1 Details on data analysis and pre-analysis plan

In the following, we discuss the details of our data analysis, the pre-analysis plan (PAP) and how we followed it. At the time we wrote the PAP in May 2021, the region- and age-specific vaccination strategy of the Swedish regions was not yet defined. Hence, we discussed in the PAP that we would need to adapt parts of the analysis to adequately capture Sweden's vaccination rollout. In the end, the Swedish vaccination strategy was more complex than we anticipated, with each of the 21 regions following a different strategy (see discussion in SM section 2.1.3). Hence, we had to make some adaptions to our analysis, as we discuss below. Otherwise, our analysis closely follows the PAP.

The PAP can be found here: `https://www.socialscienceregistry.org/trials/7652`

2.1.1 Further details on sample, outcomes, and control variables

Pre-registered data collection and sample size

Data collection: We aimed to collect data from about 10,000 participants. However, we pre-registered that there is some uncertainty about the number of people that the Survey company would be able to recruit, and that we could end up with a smaller sample size. Our instructions to the survey company were to recruit as many participants aged 18-49 as they could. At the end of data collection, the survey company was able to recruit 9,560 participants (see the Methods and Material section for details). Importantly, we had no access to the data from the vaccination registry until data collection was finished: The survey company finished data collection on July 13 and the trial data was then matched by the Public Health Agency of Sweden to population-wide administrative records of COVID-19 vaccinations on August 13. We also pre-registered that we would field the survey in age-group-specific waves based on the vaccination rollout across Sweden, such that we reached people around the time vaccination appointments opened for them. The design followed exactly the pre-registred plan (see SM Section 1).

Exclusion criteria: We pre-registered that we would exclude individuals aged <18 and >=50 and individuals for whom the Public Health Agency of Sweden recommended against getting vaccinated at the time of the trial because of heightened health risks. We excluded all these participants from the trial. To exclude individuals whom the Public Health Agency of Sweden recommended

against getting vaccinated, we asked participants on the first screen of the study whether they already received a COVID-19 vaccination, were pregnant, had previously experienced an allergic reaction that required hospital care, or had previously experienced a severe reaction after they got a vaccine. Participants who agreed to any of these statements were not allowed to participate in the study. We further excluded participants who completed the survey but whose reported social security number did not match the vaccination registries, who according to the administrative records had already received a first shot before completing the survey, who did not finish the questionnaire, and who completed the survey more than once and were assigned to different treatments (see SM Section 1).

Power for comparisons of treatment conditions to the control condition: We used simulations for power calculation. Our goal was to have enough power to compare each treatment condition to the control condition. We pre-registered that we would assign 1/3 of the participants to the control condition and 2/15 participants to each other condition. With 10,000 observations and under the assumption of a baseline in which 70% of the participants in the control group vaccinate, we estimated to have 80% power to detect effect sizes of 4 percentage points. The assumption on vaccination rates turned out to be very accurate, with 72% of participants deciding to vaccinate in the control group.

Definition of variables

We constructed our primary and secondary outcome variables and the control variables as follows:

Primary outcome variables:

- Vaccination Intention: Response to the question "Do you think you will get a first shot of a COVID-19 vaccine within the first month after the vaccine becomes available to you?" (Collected as No/Yes and coded as 0/100)
- Vaccination Uptake: Did the participant get a first shot of a COVID-19 vaccine within 30 days after he/she completed the survey? (Coded as 0/100)

Secondary outcome variables:

- Appointment Link Click: Did the participant click the link to get information on how to make a vaccination appointment? (0/1, coded as 0/100)
- Days to vaccination: How many days did the participant take to get a first shot of a COVID-19 vaccine? (coded as the number of days; censored for people who did not vaccinate)

- Vaccinated in 50 days: We pre-registered that, depending on the time window we finally observe, we might also look at whether the participant got a first shot of a COVID-19 vaccine at all within the time window we observe (secondary outcome). Since we finished the data collection of the trial on July 13 and linked the data to administrative records on August 13, we only observe vaccination uptake in a time window of 30 days for each participant. Hence, this variable turned out to be similar to our main outcome measure, Vaccination Uptake. However, for a subset of 4,181 participants, we ended up observing a time window of 50 days. For this subset, we also provide results on vaccination uptake within 50 days. (Coded as 0/100)

- Vaccination Intention Continuous: Response to the question "From 0% to 100%, what do you think are the chances that you will choose to get a first shot of a COVID-19 vaccine within the first month after the vaccine becomes available to you?" (Response scale: slider from 0% to 100%, coded as 0 to 100)

- Vaccination Intention Time: Response to the question "When do you think you will get a COVID-19 vaccine after the vaccine becomes available to you?" (Response scale: within 1 week, within 2 weeks, within 3 weeks, within 1 month, within 2 months, within 3 months, within 6 months, within 12 months, after 12 months, never, coded as 1 to 10)

- Intention-behavior gap: Vaccination Intention - Vaccination Uptake. (Coded as Vaccine Intention - Vaccination Uptake for the participants who intended to vaccinate: 100 if the participant intended to vaccinate, but did not follow-through; 0 if the participant intended to vaccinate and did vaccinate; or missing if the participant did not intend to vaccinate)

Control variables:

- Age: We pre-registered that we would select age controls as best as possible to capture the region- and age-specific vaccination strategy of Sweden and the timing of the survey waves. We pre-registered that we would construct 5-year age groups, which is what we did in our final analysis. For our analysis, we first construct an indicator for each age group capturing the following ages: 18-19, 20-24, 25-29, 30-34, 35-39, 40-44, or 45-49. In all regressions with pre-registered controls, we then control for a fixed effect for each of those age groups and for each region. We also control for the 161 interactions of each region fixed effect with each age-group fixed effect. This strategy captures the rollout strategy across Sweden quite

well, since many regions opened vaccinations gradually going from one 5-year age group to the next. However, some regions rolled out vaccinations in different steps, e.g., using age 32 as the cutoff. To address the concern that our strategy does not capture the rollout accurately, we also use specifications where we include one age effect for each age, that is, 1 fixed effect for age 19, age 20, age 21, age 22, etc. and then interact each age-specific fixed effect with the fixed effect for each region. Accounting for the resulting 736 age x region fixed effects does not affect our estimates, see SM Section 2.4.1.

- Gender: dummies for the categories indicating male/female.
- Region: dummies for each of the 21 counties in Sweden.
- Being in a COVID-19 risk group: dummy for the category "yes."
- Civil status dummies for each status: single, sarbo, couple, married, others.
- A dummy for whether children live in the participant household: dummy for number of children in the household > 0.
- Employment status dummies for each status: full-time, part-time, work, unemployed, student, pensioner, others.
- Educational attainment dummies for each group: elementary, high-school, professional training, ongoing university studies, university studies, research studies.
- Parental place of birth dummies: dummies for each place of origin of the mother and the father. (Sweden, Another European country, North America, South America, Africa, Middle-east, Rest of Asia, Oceania)
- Income dummies for each category of incomes used in the survey. (0-5000kr, 5001-10000kr, 10001-15000kr, 15001-20000kr, 20001-25000kr, 25001-30000kr, 30001-35000kr, 35001-40000kr , 40001-45000kr, 45001-50000kr, 50000kr-55000kr, more than 55000kr, coded as the midpoint value for the values below 55000kr and for the ones above as 60000kr)

Other variables:

- Economic preferences and personality traits: Altruism, Risk-affinity, Patience, Reciprocity, Trust, Procrastination, and Norm-adherence. All measures were collected on a scale from

1 to 11, with higher values indicating e.g., participants being more altruistic in the case of altruism. As pre-registered all variables are used untransformed for data analysis.

- COVID-19 related variables: Ever tested positive for COVID-19 (yes/no, 1/0), COVID-19 vaccines are safe (scale from 1 to 5), diseases can be triggered by vaccinations (scale from 1 to 5), worries about side-effects from COVID-19 vaccination (scale from 1 to 5), worries/fear of needles used for vaccination (scale from 1 to 5). As pre-registered all variables are used untransformed for data analysis. For details on all of these variables, see the experimental instructions in SM Section 2.10.

We constructed all variables exactly as pre-registered. The only deviation is the way we defined Vaccination Uptake. We initially planned to look at whether the participants got a first shot of a COVID-19 vaccine within 30 days after the vaccine became *available* to them. The rollout was substantially more complex than we expected (see discussion in SM section 2.1.3). Although we managed to recover much of the regional rollout policies through news feeds from the regional public health authorities and local newspapers, our measure on when the vaccine was available for each participant is imperfect (Vaccination Uptake Availability). Hence, before matching the data with the vaccination registries, we decided that we would slightly deviate from the pre-analysis plan and instead focus in the main analysis on whether each participant took the vaccine within 30 days after survey completion (Vaccination Uptake), with the advantage of having an exact and easily interpretable measure. SM Section 2.4.4 presents results for Vaccination Uptake Availability; all results are very similar as those provided in the main text, both in terms of effect sizes and significance. Moreover, Vaccination Uptake and Vaccination Uptake Availability are highly correlated (corr=0.88).

2.1.2 Summary of preregistered hypotheses and specification

Pre-registered main analysis

We pre-registered that we would compare Vaccination Intention and Vaccination Uptake in all treatment conditions to the control condition. We pre-registered using ordinary least squares (OLS) regressions with heteroscedasticity-robust standard errors, controlling for gender dummies, age dummies, region dummies, interactions between age and region, being in an at-risk group for COVID-19, civil status dummies, a dummy for children in the household, dummies for employment status, dummies for education, dummies for parents' place of birth, and income, as defined

above. We pre-registered using two-sided tests to examine whether treatment effects are statistically significantly different from zero. We report the results from this main analysis in the main text and in SM Section 2.3.1.[3]

We pre-registered that, as a secondary analysis, we would also look at the impact of the experimental conditions on Appointment Link Click, Intention Continuous, Intention Date, Intention-behavior Gap, and Days to Vaccination. In the main text we focus on the main outcome measures, but the findings are similar for the secondary outcome measures (SM Section 2.3.2).

Lastly, we pre-registered that we would explore whether some treatments are more effective than others. We report these results in the main text.

In the main analysis, we use the following regression specifications to estimate treatment effects:

$$\begin{aligned} VaccinationOutcome_{it} = {} & \beta_1 \times incentives_{it} + \beta_2 \times nudge_{it} + \\ & \beta_3 \times noreminders_{it} + \alpha_{age} \times \delta_{region} + X_i\gamma' + e_{it} \end{aligned} \tag{1}$$

$$\begin{aligned} VaccinationOutcome_{it} = {} & \beta_1 \times incentives_{it} + \beta_2 \times socialimpact_{it} + \\ & \beta_3 \times argument_{it} + \beta_4 \times information_{it} + \beta_5 \times noreminders_{it} + \\ & \alpha_{age} \times \delta_{region} + X_i\gamma' + e_{it} \end{aligned} \tag{2}$$

Where $VaccinationOutcome_{it}$ is the outcome of interest for participant i in treatment t and $nudge_{it}$ captures whether a person is in the social impact, argument, or information condition. The impact of each treatment relative to the control condition is captured by the corresponding β coefficient. α_{age} denotes age-group specific fixed effects, while δ_{region} denotes region specific fixed effects as pre-registered. X_i is the pre-registered vector of controls.

Pre-registered secondary analysis

Heterogeneous treatment effects according to economic preferences, personality traits, beliefs, and knowledge: We pre-registered that we would also study whether there are hetero-

[3]We deviated from the pre-analysis plan in that we pre-registered that we would provide the results of the no-reminders condition in a separate section, given that, unlike all other conditions, it did not include reminders and might therefore have decreased vaccination uptake. However, when writing the paper we thought it would be clearer if we show the results jointly. All results in terms of effect sizes and significance trivially hold whether we include the no-reminders condition or not.

geneities in treatment effects for people with different economic preferences, personality traits, vaccine beliefs, and vaccine knowledge. We noted that we would do so by regressing Vaccination Uptake on the experimental condition dummies and the interaction between treatment dummies and the respective variable (OLS with heteroscedasticity-robust standard errors and the same controls as for our main analysis).

We pre-registered that we were particularly interested in Altruism, Patience and Risk-affinity, but that we also would look at Reciprocity, Trust, Present focus, Norm-adherence, Vaccination attitudes and the rest of the variables (e.g., gender, education, parental place of birth, income, COVID-19 history, fear of needles, etc.). We provide all these results in SM Section 2.5. We do not find robust heterogeneities; instead, incentives increase vaccination rates similarly for all subgroups.

We pre-registered that we also would study whether the individual characteristics that we measure can be used to improve the effectiveness of the interventions by targeting interventions to participants. Given that this approach would rely on the existence of heterogeneous treatment effects, which we did not find in the data, we did not follow through with this initial plan.

Correlations between vaccination uptake and preferences, personality traits, and COVID-19/vaccination related variables: We pre-registered that we would also study how economic preferences, personality traits, vaccine beliefs, and vaccine knowledge are related to vaccine behaviors and the intention-behavior gap.

We pre-registered to only focus on the data in the Control and no-reminders (Minimal) condition and regress (OLS with heteroscedasticity-robust standard errors and same controls as for our main analysis) Vaccination Uptake and the Intention-behavior gap on different individual characteristics, preferences, personality traits, vaccine beliefs, and vaccine knowledge: Altruism, Risk-affinity, Patience, Beliefs about COVID-19 vaccine risk, Reciprocity, Trust, Procrastination, Norm following, Vaccine knowledge, Worries about COVID-19 vaccination side effects, and Fear of needles. In addition, we pre-registered that we would explore whether there are relevant individual differences using the rest of the variables (e.g. gender, education, parental place of birth, income, COVID-19 history, etc.). Variables for balance checks and heterogeneity analyses where the coding was not pre-registered: College degree (yes/no, at least currently enrolled in a college), Foreign (both parents were not born in Europe and at least one parent was born outside of Europe). We indicated that we would report this analysis both for each characteristic separately and adding measures jointly. We show all these results in SM Section 2.6.

2.1.3 Details on the Swedish vaccination rollout and timing of the experiment

Vaccination against COVID-19 in Sweden is administered by 21 regional health authorities (henceforth referred to as regions), coordinated by the Public Health Agency of Sweden through recommendations about priority groups and other aspects of the vaccine campaign. The vaccine was offered to all adults living in Sweden free of charge. For the age groups that our participants belong to (18-49), only the Pfizer-BioNTech and Moderna vaccines were used. While the Swedish government has not officially stated what percentage of the population it aims to vaccinate, the authorities have repeatedly conveyed that they recommend every eligible adult to get vaccinated. We hence believe that it is safe to assume that the Swedish authorities hope that as many people vaccinate as possible.

Sweden rolled out the vaccine in four phases. In the first phase, starting in December 2020, vaccinations were offered to individuals living in elderly-care homes, their close contacts, and elderly-care healthcare personnel. In the second and third phases, initiated in March 2021, vaccinations were administered to individuals above 60 years, healthcare workers, and adults belonging to risk groups. The fourth phase, which begun in May 2021, opened up vaccinations to all adults (18+), starting with the oldest groups.

The vaccination was organized by the 21 regions. The dates when the vaccine became available to different age groups varied across regions, and due to sudden shocks to the supply of vaccines, the schedule was frequently revised. The date a person could book a vaccination slot differed by up to a month, depending on the region. While many regions gradually opened for new groups using 5-year intervals or finer, other regions used larger age intervals for the rollout. Some regions were alternating and opening vaccination appointments moving from one specific age to the next within a matter of days. The details of the complex rollout have not been well documented. However, we were able to recover many of the specifics through news feeds from the regional public health authorities and local newspapers, although our measure is imperfect. Table S1 displays our approximation of the dates when the booking of the vaccines opened across the 21 regions by age group.

Figure S2 describes the timing of the vaccine rollout in relation to the date of participation in our experiment, based on our imperfect measure of availability in each region and age category. While the starting dates varied across regions, the survey invitations were sent to participants across all regions simultaneously. Therefore, for respondents in some regions, the booking system had already been open for some time when they received their invitations, while others had to wait until

vaccinations started in their region. The median participant filled in the survey one day before the vaccination booking site opened for his/her region and age group. It should be noted that during the first days after vaccination opened to a new age group, the demand for vaccines typically exceeded supply, implying that participants likely would need wait up to two weeks before finding an available slot. However, people could typically book an appointment within at most two weeks. In some cases, participants could book vaccine slots in other regions, but we observe that this is rare. Only 61 of the participants got the vaccine outside of their region of residence.

The vaccination data from the regions are sent to the Public Health Agency of Sweden and included in the National Vaccination Register (Nationella vaccinationsregistret). The vaccination data we use was extracted by the Public Health Agency of Sweden on August 13 and linked to our trial data by the Public Health Agency of Sweden. Figure S3, displays the number days between the survey and the matching with the vaccination data. The three waves of the survey are apparent in the data and, with the exception of a 28 participants, we observe more than 30 days between survey participation and the matching of the data for all participants.

Table S1: Table on vaccination rollout dates

Region	Ages<br>18-24	25-29	30-34	35-39	40-44	45-49
Stockholm	09.07.2021	29.06.2021	29.06.2021	14.06.2021	03.06.2021	21.05.2021
Uppsala	14.07.2021	25.06.2021	22.06.2021	15.06.2021	08.06.2021	04.06.2021
Sörmland	22.06.2021	18.06.2021	15.06.2021	11.06.2021	04.06.2021	27.05.2021
Östergötland	07.07.2021	02.07.2021	29.06.2021	24.06.2021	17.06.2021	10.06.2021
Jönköpings län	30.06.2021	30.06.2021	15.06.2021	15.06.2021	04.06.2021	04.06.2021
Kronoberg	28.06.2021	28.06.2021	28.06.2021	28.06.2021	21.06.2021	14.06.2021
Kalmar	01.07.2021	01.07.2021	21.06.2021	21.06.2021	10.06.2021	10.06.2021
Gotland	09.07.2021	07.07.2021	28.06.2021	21.06.2021	16.06.2021	09.06.2021
Blekinge	06.07.2021	06.07.2021	16.06.2021	16.06.2021	28.05.2021	28.05.2021
Skåne	06.07.2021	30.06.2021	23.06.2021	15.06.2021	08.06.2021	04.06.2021
Halland	02.07.2021	28.06.2021	22.06.2021	15.06.2021	10.06.2021	01.06.2021
Västra Götaland	30.06.2021	30.06.2021	30.06.2021	30.06.2021	22.06.2021	08.06.2021
Värmland	28.06.2021	23.06.2021	21.06.2021	21.06.2021	11.06.2021	07.06.2021
Örebro	02.07.2021	28.06.2021	24.06.2021	19.06.2021	08.06.2021	07.06.2021
Västmanland	01.07.2021	01.07.2021	01.07.2021	22.06.2021	15.06.2021	08.06.2021
Dalarna	29.06.2021	17.06.2021	01.06.2021	01.06.2021	01.06.2021	01.06.2021
Gävleborg	05.07.2021	29.06.2021	15.06.2021	15.06.2021	01.06.2021	25.05.2021
Västernorrland	28.06.2021	28.06.2021	28.06.2021	09.06.2021	09.06.2021	09.06.2021
Jämtland Härjedalen	08.07.2021	08.07.2021	01.07.2021	01.07.2021	20.05.2021	20.05.2021
Västerbotten	09.07.2021	09.07.2021	24.06.2021	24.06.2021	18.06.2021	11.06.2021
Norrbotten	11.06.2021	11.06.2021	11.06.2021	11.06.2021	03.06.2021	03.06.2021

Figure S2: Distribution of the days between trial participation and vaccination rollout in a participants' region

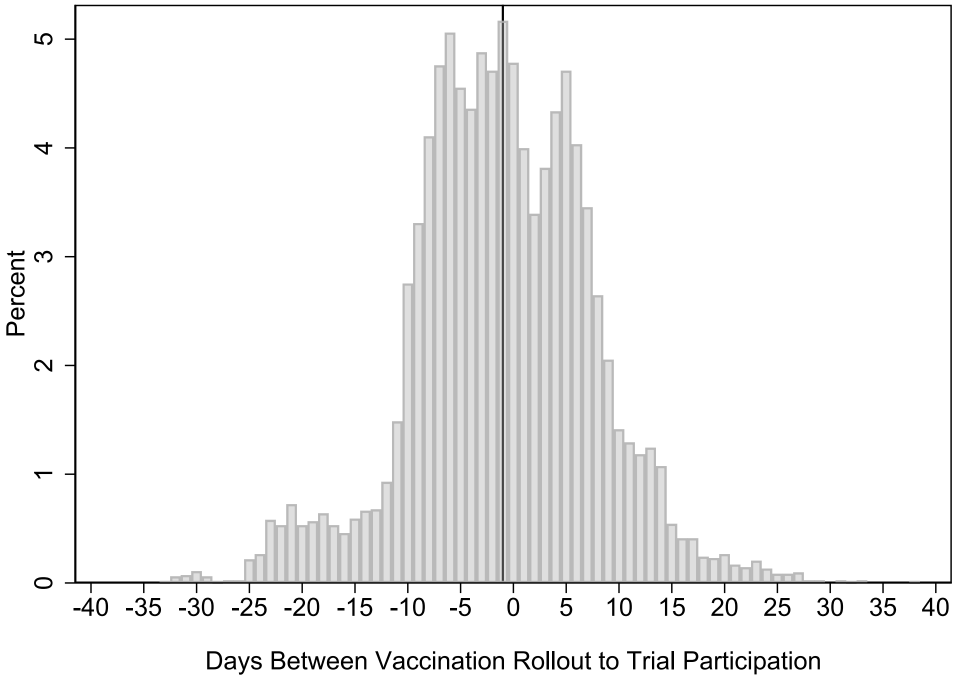

Figure S3: Distribution of observed days between trial participation and linkage with administrative records

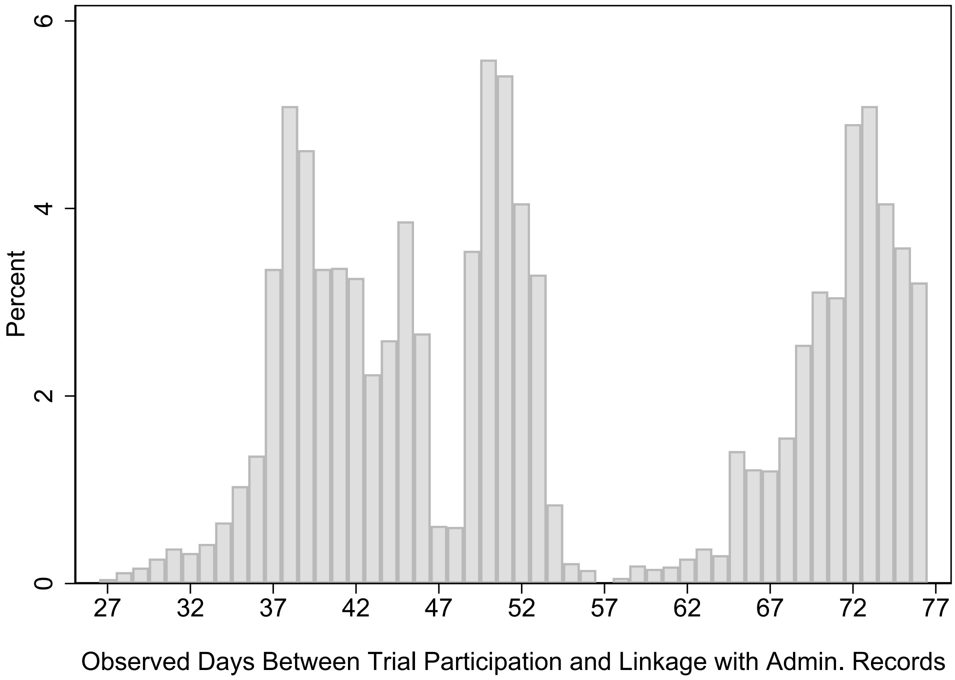

2.2 Summary statistics, balance, and sample

In this section, we provide an overview of the data. We first provide summary statistics of all of our outcome and explanatory variables. Next, we study whether these variables are well-balanced across the different treatment conditions. Finally, we use data from Statistics Sweden to study the representativeness of our sample.

2.2.1 Summary statistics

Table S2 below provides descriptive statistics of our sample. The table first reports the summary statistics for our outcome variables, including people's behavior and intentions. We then report the proportion randomly assigned to each treatment, which as planned corresponds to 2/15 of the participants to each treatment and 5/15 to the control condition. We then report the summary statistics for the background variables gathered in the survey, including participants' sociodemographics, answers to vaccine and COVID-19 specific questions, and economic preferences and personality.

Table S2: Summary Statistics

Variable	Mean	SD	Min.	Max.	N
Behaviors and Intentions					
Vaccinated Within 30 days	72.85	44.48	0	100	8,286
Days Between Vaccination and Survey	14.69	10.96	0	74	6,582
Appointment Link Click	5.50	22.81	0	100	8,286
Intention to Vaccinate	84.87	35.84	0	100	8,286
Vaccination Int. Continuous	82.11	29.26	0	100	8,261
Vaccination Int. Timing	3.18	2.44	1	10	8,249
Intention-Behavior Gap	17.78	38.23	0	100	7,032
Treatment Assignment					
Incentives Cond.	0.14	0.34	0	1	8,286
All Nudges	0.39	0.49	0	1	8,286
Social Impact Cond.	0.13	0.34	0	1	8,286
Argument Cond.	0.13	0.34	0	1	8,286
Information Cond.	0.13	0.34	0	1	8,286
No reminders Cond.	0.13	0.34	0	1	8,286
Control Cond.	0.34	0.47	0	1	8,286
Sociodemographics					
Age	34.61	8.34	18	49	8,286
Female	0.58	0.49	0	1	8,286
Single	0.26	0.44	0	1	8,286
Sarbo	0.06	0.23	0	1	8,286
Couple	0.35	0.48	0	1	8,286
Married	0.31	0.46	0	1	8,286
Other Civil Status	0.02	0.15	0	1	8,286

Continued on next page

Table S2 – *Continued from previous page*

Variable	Mean	SD	Min.	Max.	N
Has Children	0.53	0.50	0	1	8,286
Elementary School or Lower	0.02	0.15	0	1	8,286
High-school	0.30	0.46	0	1	8,286
Professional Training	0.13	0.33	0	1	8,286
In College	0.07	0.26	0	1	8,286
College Degree	0.46	0.50	0	1	8,286
PhD	0.02	0.13	0	1	8,286
Employed	0.81	0.39	0	1	8,286
Unemployed	0.04	0.18	0	1	8,286
In College	0.11	0.31	0	1	8,286
Retired	0.01	0.08	0	1	8,286
Other Professional Situation	0.04	0.20	0	1	8,286
Mother from Sweden	0.85	0.35	0	1	8,286
Mother from Rest of Europe	0.10	0.30	0	1	8,286
Mother from North America	0.00	0.05	0	1	8,286
Mother from South America	0.01	0.09	0	1	8,286
Mother from Africa	0.00	0.06	0	1	8,286
Mother from the Middle-east	0.02	0.13	0	1	8,286
Mother from the Rest of Asia	0.01	0.12	0	1	8,286
Mother from Oceania	0.00	0.02	0	1	8,286
Father from Sweden	0.85	0.36	0	1	8,286
Father from Rest of Europe	0.10	0.30	0	1	8,286
Father from North America	0.00	0.06	0	1	8,286
Father from South America	0.01	0.09	0	1	8,286
Father from Africa	0.01	0.08	0	1	8,286
Father from the Middle-east	0.02	0.15	0	1	8,286
Father from the Rest of Asia	0.01	0.11	0	1	8,286
Father from Oceania	0.00	0.02	0	1	8,286
Income 0-5000kr	0.03	0.18	0	1	8,286
Income 5001-10000kr	0.05	0.22	0	1	8,286
Income 10001-15000kr	0.11	0.31	0	1	8,286
Income 15001-20000kr	0.11	0.31	0	1	8,286
Income 20001-25000kr	0.22	0.41	0	1	8,286
Income 25001-30000kr	0.20	0.40	0	1	8,286
Income 30001-35000kr	0.13	0.34	0	1	8,286
Income 35001-40000kr	0.07	0.26	0	1	8,286
Income 35001-40000kr	0.04	0.18	0	1	8,286
Income 45001-50000kr	0.02	0.13	0	1	8,286
Income 50000kr-55000kr	0.01	0.10	0	1	8,286
Income more than 55000kr	0.01	0.12	0	1	8,286
Economic Preferences and Personality Traits					
Altruism	8.51	2.40	1	11	8,286
Risk-affinity	6.67	1.93	1	11	8,286
Patience	8.02	1.77	1	11	8,286
Reciprocity	9.88	1.39	1	11	8,286
Trust	6.96	2.24	1	11	8,286

Continued on next page

Table S2 – *Continued from previous page*

Variable	Mean	SD	Min.	Max.	N
Procrastination	6.57	2.67	1	11	8,286
Norm-adherence	7.57	2.21	1	11	8,286
COVID-19 Related					
Ever tested positive for COVID-19	0.22	0.42	0	1	8,286
COVID-19 vaccines are safe	4.13	1.04	1	5	8,286
Diseases can be triggered by vaccinations	1.93	1.06	1	5	8,286
Worried about side-effects	2.60	1.36	1	5	8,286
Worried about needles	2.21	1.43	1	5	8,286

2.2.2 Balance

We randomly assigned participants to the experimental conditions. Table S3 tests whether participants' main sociodemographic variables are balanced across treatment conditions. To do so, we perform OLS regressions explaining each of the sociodemographic variables with each of the treatment conditions (without any controls). We then report the coefficient, standard errors, and significance level of each treatment condition. We do not see substantial differences across treatment conditions, neither in the eight reported sociodemographics nor including any of the other background variables. This implies that randomization was indeed successful in balancing the different treatment conditions.

Table S3: Balance checks

Dependent Variable	Age		Female		Single		Has childr.		College		Income		Immigr.		Unemployed	
	(1)	(2)	(3)	(4)	(5)	(6)	(7)	(8)	(9)	(10)	(11)	(12)	(13)	(14)	(15)	(16)
Incentives Cond.	0.24	0.24	0.02	0.02	0.00	0.00	-0.01	-0.01	0.01	0.01	-0.01	-0.01	-0.01	-0.01	0.01	0.01
	(0.29)	(0.29)	(0.02)	(0.02)	(0.02)	(0.02)	(0.02)	(0.02)	(0.02)	(0.02)	(0.04)	(0.04)	(0.01)	(0.01)	(0.01)	(0.01)
All Nudges	0.11		0.00		-0.02		-0.00		0.03**		0.04		-0.01		-0.00	
	(0.22)		(0.01)		(0.01)		(0.01)		(0.01)		(0.03)		(0.01)		(0.00)	
Social Impact Cond.		0.03		0.02		-0.03*		0.01		0.02		0.02		-0.01*		-0.00
		(0.30)		(0.02)		(0.02)		(0.02)		(0.02)		(0.04)		(0.01)		(0.01)
Argument Cond.		0.22		-0.01		-0.01		0.00		0.03*		0.08**		-0.00		-0.01
		(0.30)		(0.02)		(0.02)		(0.02)		(0.02)		(0.04)		(0.01)		(0.01)
Information Cond.		0.07		0.01		-0.01		-0.03		0.02		0.02		-0.01		0.00
		(0.30)		(0.02)		(0.02)		(0.02)		(0.02)		(0.04)		(0.01)		(0.01)
No Reminders Cond.	0.36	0.36	-0.02	-0.02	-0.02	-0.02	0.03*	0.03*	0.03*	0.03*	0.05	0.05	-0.01	-0.01	-0.01*	-0.01*
	(0.29)	(0.29)	(0.02)	(0.02)	(0.02)	(0.02)	(0.02)	(0.02)	(0.02)	(0.02)	(0.04)	(0.04)	(0.01)	(0.01)	(0.01)	(0.01)
Observations	8286	8286	8286	8286	8286	8286	8286	8286	8286	8286	8286	8286	8286	8286	8286	8286

Note: Results from an OLS regression in which we explain each socioeconomic variable with each of the five different conditions. Income in 10,000 SEK. Heteroscedasticity robust standard errors in parentheses. * $p < 0.10$, ** $p < 0.05$, *** $p < 0.01$

2.2.3 General population sample

Next we compare the demographic composition of our general population sample with the Swedish population (considering the same age restriction). The survey company can only provide a sample that is representative in terms of age, gender, and region. However, we can nevertheless also compare other characteristics to the general population. In addition to age, gender, and region, we also document how our sample compares to the general population in terms of average income, education, and immigration background.

We obtained the Swedish population data from the public registry data reported by Statistics Sweden. While the sample is similar to the general Swedish population in terms of age, region, and income, people with a college education are slightly overrepresented in our sample, immigrants are underrepresented in our sample, and women are slightly overrepresented. In SM Section 2.4.3 we show that results do not change when using sampling weights to reweight our sample.

Table S4: RCT sample and the Swedish population

**Variable**	Data from our RCT			Sweden Ages 18-49
	N	Mean	SD	Mean
Age	8,286	34.61	8.34	33.71
Female	8,286	0.58	0.49	0.49
Region Stockholm	8,286	0.24	0.42	0.25
Region Östra Mellansverige	8,286	0.17	0.37	0.17
Region Småland med öarna	8,286	0.07	0.26	0.08
Region Sydsverige	8,286	0.14	0.35	0.15
Region Västsverige	8,286	0.22	0.42	0.20
Region Norra Mellansverige	8,286	0.06	0.24	0.07
Region Mellersta Norrland	8,286	0.04	0.19	0.03
Region Övre Norrland	8,286	0.05	0.23	0.05
Share university education	8,286	0.48	0.50	0.44
Average monthly income (SEK)	8,286	24,847	10,951	24,211*
Both parents born in Sweden	8,286	0.80	0.40	0.60†

Note: Comparison of the trial data on age, gender, region, university education, average income, and immigration background with public registry data from Statistics Sweden. We constructed the variables such that they match the Statistics Sweden definition, with two exceptions: * is based on age >19, † is based on both parents were born in Sweden *and* the individual was born in Sweden (due to privacy concerns, we did not elicit whether the individual was born in Sweden in our survey).

2.3 Main results and robustness

In this section, we provide the key tables and figures that are not reported in the main text. When not mentioned otherwise, all the tables correspond to the pre-registered specification described in SM Section 2.1.2, i.e. OLS regressions with heteroscedasticity-robust standard errors. The controls also correspond to the pre-registered controls specified in SM Section 2.1.2. The coefficient for All Nudges corresponds to an indicator variable which captures whether a participant was part of the social impact, the argument, or the information condition.

2.3.1 Main outcome variables

Table S5: Treatment effects on vaccination uptake and intentions. This table provides the effect sizes from our main specification with the pre-registered set of controls (SM Section 2.1.2). The table shows that incentives increase both vaccination uptake (whether a participant vaccinates within 30 days after filling out the survey) and vaccination intentions (whether a participant intends to get the vaccine within 30 days). While some behavioral nudges seem to impact vaccination intentions, none of them have any statistically significant effects on vaccination uptake.

Table S5: Treatment effects on vaccination uptake and intentions

Dependent Variable	Vaccination Uptake		Vaccination Intentions	
	(1)	(2)	(3)	(4)
Incentives Cond.	4.18***	4.18***	3.72***	3.71***
	(1.50)	(1.50)	(1.20)	(1.20)
All Nudges	1.17		1.77*	
	(1.14)		(0.93)	
Social Impact Cond.		1.42		2.22*
		(1.55)		(1.23)
Argument Cond.		1.35		2.72**
		(1.56)		(1.24)
Information Cond.		0.74		0.35
		(1.58)		(1.30)
No reminders Cond.	-0.95	-0.95	0.21	0.21
	(1.58)	(1.58)	(1.29)	(1.29)
Age x Region FE	yes	yes	yes	yes
Controls	yes	yes	yes	yes
Observations	8,286	8,286	8,286	8,286

Note: The table shows coefficient estimates from linear regressions of the outcome variable on each of the treatment conditions of the treatment conditions. All nudges pool the *social impact*, *argument*, and *information condition*. Heteroscedasticity robust standard errors are shown in parentheses. All regressions use the pre-registered controls: gender, age, region, interactions between age and region, being in an at-risk group for COVID-19, civil status, having children in the household, employment status, education, parents' place of birth, and income. See SM Section 2.1.1 for the pre-registered definitions of each control and outcome variable.
* $p < 0.10$, ** $p < 0.05$, *** $p < 0.01$

Figure S4: Vaccination uptake by experimental condition. This figure represents the raw vaccination uptake rates of each of the experimental conditions. Table S5 uses the pre-registered analysis to test whether the observed differences between each of the treatments and the control condition are statistically significant.

Figure S4: Vaccination uptake by experimental condition

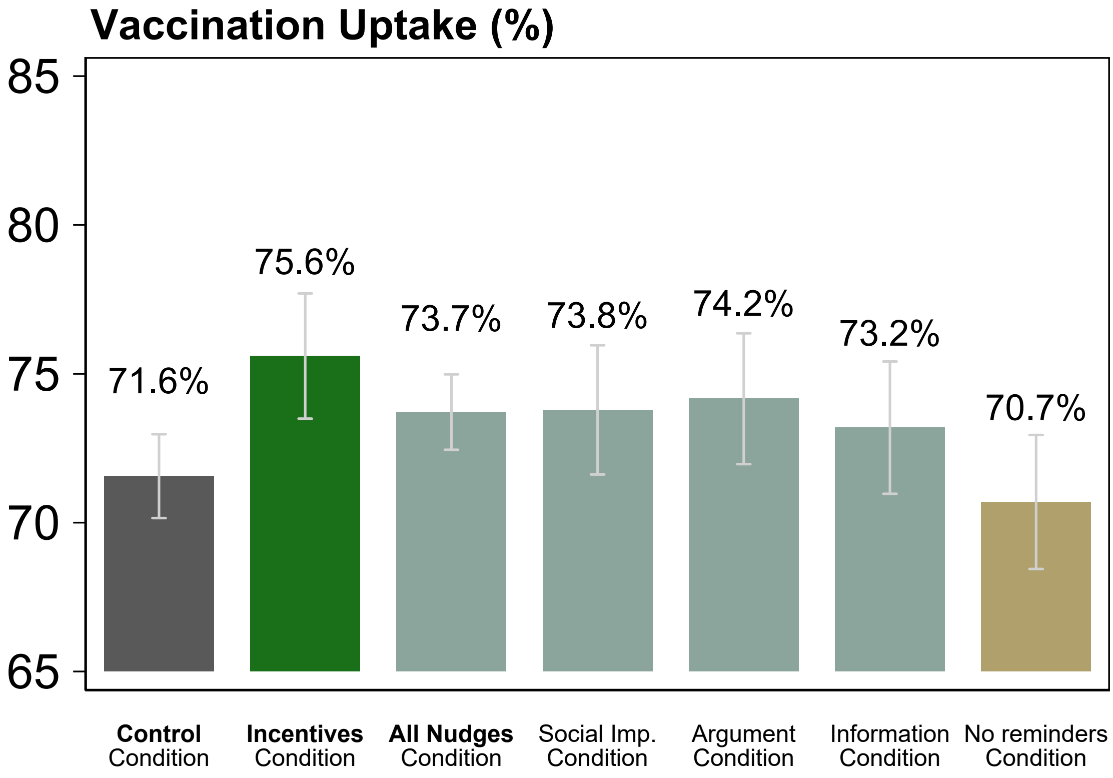

Figure S5: Vaccination intentions by experimental condition. This figure represents the raw vaccination intentions rates of each of the experimental conditions. Table S5 uses the pre-registered analysis to test whether the observed differences between each of the treatments and the control condition are statistically significant.

Figure S5: Vaccination intentions by experimental condition

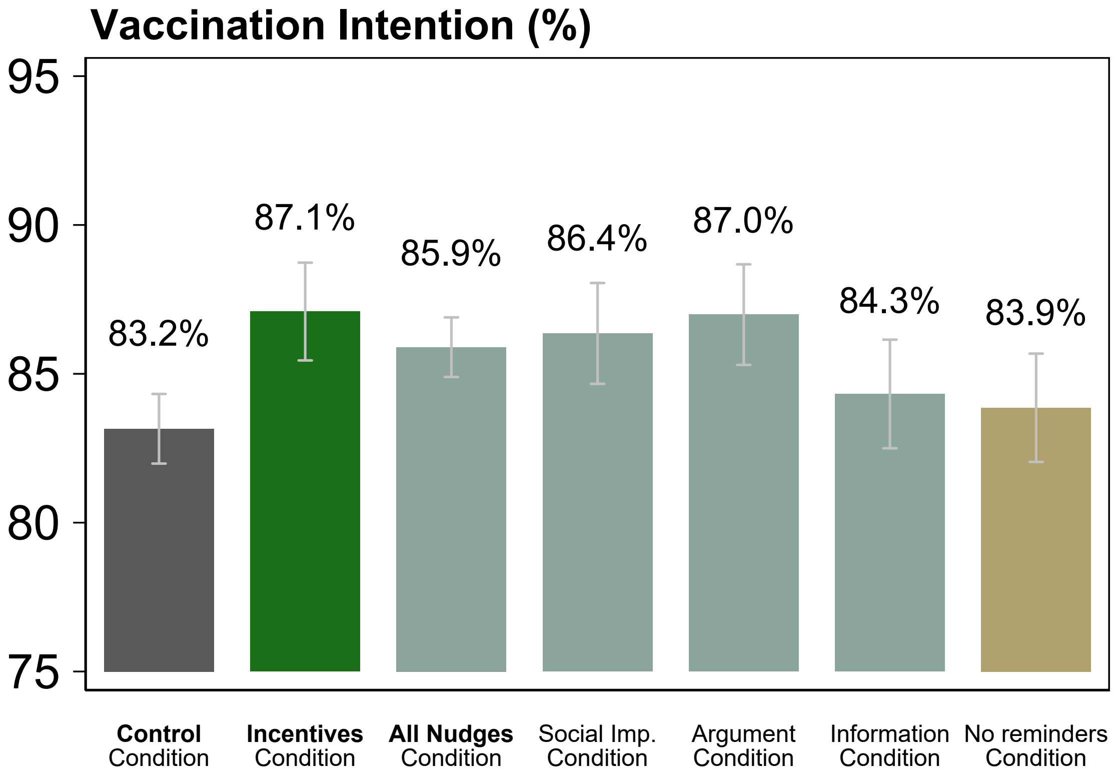

2.3.2 Secondary outcome variables

Table S6: Treatment effects using the secondary outcome variables. This table studies the treatment effects on the pre-registered secondary outcome variables described in SM Section 2.1.2 (except the variable "Vaccinated in 50 days" which is studied in detail in the next table, SM Section 2.3.3). By using the pre-registered controls described in SM Section 2.1.2, we study the regression-estimated impacts of the experimental conditions on Days to vaccinate (the number of days that it takes for the participant to vaccinate after filling out the survey), Appointment Link Click (whether participants click on the appointment link at the end of the survey), Intention Continuous (the probability that participants assign to getting the vaccine within 30 days), Intention Time (within how many days participant say that they intend to get the vaccine), and the Intention-Behavior gap (which is coded as 100 if the participant intends to vaccinate but does not follow through, 0 if the participant intends to vaccinate and follows through, and missing if the participant does not intend to vaccinate). Column (1) performs a Tobit regression, accounting for the fact that our observations are censored. The other columns use OLS as described in SM Section 2.1.2. All regressions use the pre-registered set of controls.

The table shows that incentives robustly make participants vaccinate earlier, more likely to click the appointment link, state that they are more likely to get the vaccine, and that they intend to get the vaccine sooner. The rest of the conditions have little impact, with the exception of the argument condition which affects participants' stated probability to get the vaccine. The table also shows that none of the treatment conditions affects the intention-behavior gap.

Table S6: Treatment effects using the secondary outcome variables

Dependent Variable	Days to Vaccination		Appointment Link Click Yes=100 or No=0		Intention Measure: Continuous 0 to 100%		Intention Measure: Time 2 to 10		Intention-behavior Gap Yes=100 or No=0	
	(1)	(2)	(3)	(4)	(5)	(6)	(7)	(8)	(9)	(10)
Incentives Cond.	-1.27***	-1.27***	4.92***	4.92***	2.11**	2.10**	-0.17**	-0.17**	-1.70	-1.70
	(0.47)	(0.47)	(1.00)	(1.00)	(0.95)	(0.95)	(0.08)	(0.08)	(1.42)	(1.42)
All Nudges	-0.27		-0.04		1.18		-0.05		-0.58	
	(0.34)		(0.60)		(0.75)		(0.06)		(1.07)	
Social Impact Cond.		-0.22		0.90		0.46		-0.02		0.00
		(0.47)		(0.86)		(1.01)		(0.08)		(1.47)
Argument Cond.		-0.33		0.24		2.02**		-0.09		-0.46
		(0.48)		(0.84)		(1.00)		(0.09)		(1.46)
Information Cond.		-0.27		-1.30*		1.10		-0.05		-1.32
		(0.48)		(0.77)		(1.06)		(0.09)		(1.47)
No reminders Cond.	0.40	0.40			-2.02*	-2.02*	0.11	0.11	0.41	0.41
	(0.48)	(0.48)			(1.06)	(1.06)	(0.09)	(0.09)	(1.49)	(1.49)
Age x Region FE	yes	yes	yes	yes	yes	yes	yes	yes	yes	yes
Controls	yes	yes	yes	yes	yes	yes	yes	yes	yes	yes
Observations	8,286	8,286	7,177	7,177	8,261	8,261	8,249	8,249	7,032	7,032

Note: Columns (3)-(10) show coefficient estimates from linear regressions of the outcome variable on each of the treatment conditions. Columns (1) and (2) show coefficient estimates from Tobit regressions to account for the fact that the data is censored. The results are equivalent when using Poisson regression or OLS regressions censoring the Days to Vaccination at 30. All nudges pool the *social impact*, *argument*, and *information condition*. Heteroscedasticity robust standard errors are shown in parentheses. All regressions use the pre-registered controls: gender, age, region, interactions between age and region, being in an at-risk group for COVID-19, civil status, having children in the household, employment status, education, parents' place of birth, and income. See SM Section 2.1.1 for the pre-registered definitions of each control and outcome variable. There are fewer observations in columns (3) and (4) since the No reminders Condition did not include an appointment link. * $p < 0.10$, ** $p < 0.05$, *** $p < 0.01$

Figure S6: Link clicking by experimental condition. This figure represents the proportion of participants who clicked the link to the vaccine appointment website after finishing the survey (Appointment Link Click). While the behavioral nudges do not seem to have much of an effect, the incentives condition increases the rates substantially. Table S9 uses the pre-registered specification to test whether these differences are statistically significant.

Figure S6: Clicks on the appointment link by experimental condition

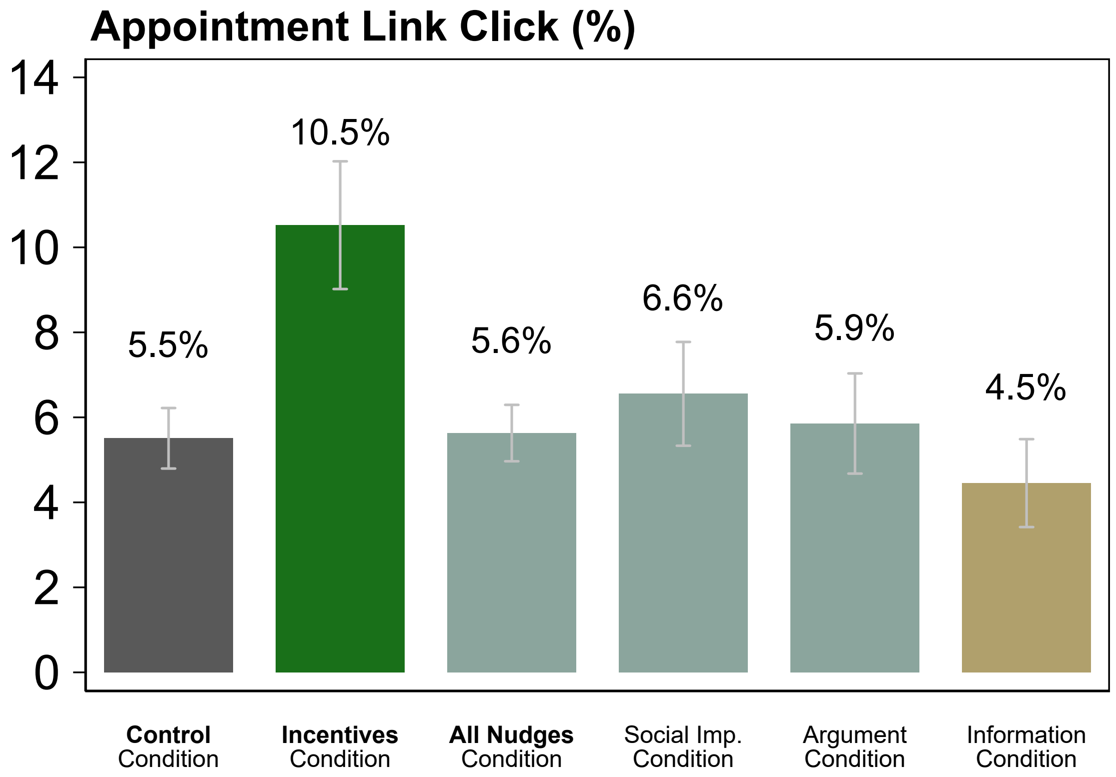

2.3.3 Considering different time windows

Table S7: Treatment effects on vaccination uptake by different time windows. The following table provides regression-estimated impacts of the experimental conditions on vaccination uptake within 50 days of survey completion. Note that we only observe vaccination uptake in a time window of 50 days for a subset of 4,181 participants. We also provide results on vaccination uptake within 10, 20, 30, and 40 days for the same sample. We observe similar impacts of incentives when considering vaccination uptake within 10, 20, 30, 40, and 50 days after survey completion. These results highlight that incentives not only accelerate vaccination uptake in the short-run, but increase uptake for at least 50 days.

Table S7: Treatment effects on vaccination uptake by different time windows

Dependent Variable	Vaccinated in 10 Days		Vaccinated in 20 Days		Vaccinated in 30 Days		Vaccinated in 40 Days		Vaccinated in 50 Days	
	(1)	(2)	(3)	(4)	(5)	(6)	(7)	(8)	(9)	(10)
Incentives Cond.	5.63***	5.63***	3.26	3.27	4.74**	4.75**	4.28**	4.28**	3.75**	3.75**
	(2.04)	(2.05)	(2.27)	(2.27)	(2.11)	(2.11)	(1.96)	(1.96)	(1.90)	(1.90)
All Nudges	2.37		0.33		1.37		1.30		1.15	
	(1.49)		(1.73)		(1.62)		(1.52)		(1.47)	
Social Impact Cond.		2.20		1.43		2.62		1.22		0.58
		(2.06)		(2.36)		(2.20)		(2.08)		(2.02)
Argument Cond.		2.48		-0.55		-0.15		1.35		1.94
		(2.08)		(2.40)		(2.23)		(2.03)		(1.93)
Information Cond.		2.44		0.08		1.62		1.34		0.92
		(2.10)		(2.43)		(2.26)		(2.09)		(2.03)
No Reminders Cond.	-1.29	-1.29	-3.82	-3.82	-1.84	-1.84	-1.01	-1.01	-1.59	-1.59
	(1.98)	(1.98)	(2.34)	(2.34)	(2.22)	(2.22)	(2.07)	(2.07)	(2.02)	(2.02)
Age x Region FE	yes	yes	yes	yes	yes	yes	yes	yes	yes	yes
Controls	yes	yes	yes	yes	yes	yes	yes	yes	yes	yes
Observations	4,181	4,181	4,181	4,181	4,181	4,181	4,181	4,181	4,181	4,181

Note: The table shows coefficient estimates from linear regressions of the outcome variable on each of the treatment conditions of the treatment conditions. The sample is restricted to those observations for whom we observe whether they vaccinated within 50 days after answering the survey. All nudges pool the *social impact*, *argument*, and *information condition*. Heteroscedasticity robust standard errors are shown in parentheses. All regressions use the pre-registered controls: gender, age, region, interactions between age and region, being in an at-risk group for COVID-19, civil status, having children in the household, employment status, education, parents' place of birth, and income. See SM Section 2.1.1 for the pre-registered definitions of each control and outcome variable.
* $p < 0.10$, ** $p < 0.05$, *** $p < 0.01$

Figure S7: Proportion of participants who got the vaccine per day after the trial. This figure plots the Kaplan-Meier curves of the proportion of participants who got the vaccine by a given day after participation in the trial. The figure shows that those in the incentives group vaccinated at a higher pace.

Figure S7: Proportion of participants who got vaccinated per day after the trial

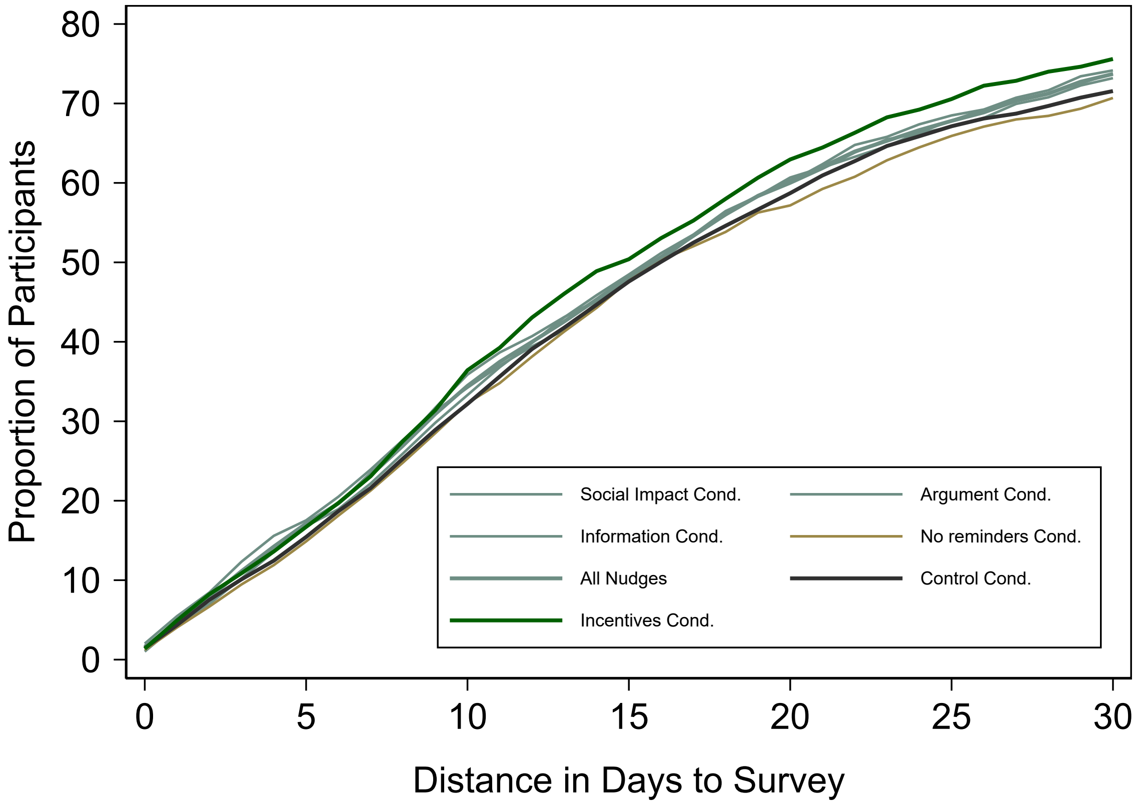

2.3.4 Alternative vaccination uptake definitions

Table S8: Treatment effects on vaccination uptake, different definitions. This table provides regression-estimated impacts of the experimental conditions on vaccination uptake using different definitions of the outcome variable. More concretely, it uses Vaccination Uptake (whether a participant got the vaccine within 30 days after participating), Vaccination Uptake Availability (whether a participant got the vaccine within 30 days after it became available to them, as proxied from the news feed of regions and local newspapers, see SM Section 2.1.3), and Vaccination Uptake Survey/Availability. The latter variable combines both previous variables, with the 30 days starting at the date of the survey if the vaccine was already available for the participant, and if the vaccine was not yet available, the 30 days started when it became available. The results indicate that the effects of the incentives condition on vaccine uptake are robust to the definition of the outcome variable. Furthermore, none of the behavioral nudges seems to have any affect on any of the outcome variable definitions.

Table S8: Treatment effects on vaccination uptake, different definitions

Dependent Variable	Uptake		Vaccination Uptake Availability		Uptake Survey/Avail.	
	(1)	(2)	(3)	(4)	(5)	(6)
Incentives Cond.	4.18***	4.18***	4.28***	4.27***	3.89***	3.89***
	(1.50)	(1.50)	(1.48)	(1.48)	(1.46)	(1.46)
All Nudges	1.17		1.61		1.20	
	(1.14)		(1.12)		(1.10)	
Social Impact Cond.		1.42		0.90		0.71
		(1.55)		(1.54)		(1.50)
Argument Cond.		1.35		2.64*		2.21
		(1.56)		(1.51)		(1.49)
Information Cond.		0.74		1.30		0.69
		(1.58)		(1.55)		(1.53)
No reminders Cond.	-0.95	-0.95	-1.07	-1.07	-0.64	-0.64
	(1.58)	(1.58)	(1.56)	(1.56)	(1.53)	(1.53)
Age x Region FE	yes	yes	yes	yes	yes	yes
Controls	yes	yes	yes	yes	yes	yes
Observations	8,286	8,286	8,286	8,286	8,286	8,286

Note: The table shows coefficient estimates from linear regressions of the outcome variable on each of the treatment conditions of the treatment conditions. All nudges pool the *social impact*, *argument*, and *information condition*. Heteroscedasticity robust standard errors are shown in parentheses. All regressions use the pre-registered controls: gender, age, region, interactions between age and region, being in an at-risk group for COVID-19, civil status, having children in the household, employment status, education, parents' place of birth, and income. See SM Section 2.1.1 for the pre-registered definitions of each control. See the text above for information on how each outcome variable is coded. * $p < 0.10$, ** $p < 0.05$, *** $p < 0.01$

2.3.5 Results comparing incentives to all nudges

Table S9: Comparing incentives to nudges on Vaccination Uptake, Vaccination Intention and Appointment Link Click. This table restricts the sample to the participants who were assigned to the incentives condition or one of the three nudge conditions (social impact, argument, and information conditions). It then shows the treatment effects of the incentives condition relative to to the 3 nudge conditions pooled on Vaccination Uptake, Vaccination Intention and Appointment Link Click (that is, whether the participant clicked on a link to the appointment website that we gave at the end of the survey). We use our main specification with the pre-registered set of controls (SM Section 2.1.2).

The table shows that incentives increase Vaccination Uptake significantly more than the three behavioral nudges together. It also shows that participants in the incentives condition are much more likely (from a base rate of 5.5%) to click on the appointment link.

The pairwise comparison show similar differences in terms of coefficient estimates, but are less precisely estimated. Differences of incentives vs. each condition: 2.67 pp (p=0.15) for the social impact condition, 3.13 pp (p=0.096) for the argument condition, and 3.16 pp (p=0.098) for the information condition.

Table S9: Comparing incentives to nudges

Dependent Variable	Vaccination Uptake	Vaccination Intentions	App. Link Click
	(1)	(2)	(3)
Incentives Cond.	3.06**	2.01*	4.80***
	(1.48)	(1.16)	(1.00)
Age x Region FE	yes	yes	yes
Controls	yes	yes	yes
Observations	4,399	4,399	4,399

Note: This table restricts the observations to those participants who were in the *incentives, social impact, argument,* and *information conditions*. The table shows coefficient estimates from linear regressions of the outcome variable on the incentives condition (thus comparing incentives to the three nudges pooled). All nudges pool the *social impact*, *argument*, and *information condition*. Heteroscedasticity robust standard errors are shown in parentheses. All regressions use the pre-registered controls: gender, age, region, interactions between age and region, being in an at-risk group for COVID-19, civil status, having children in the household, employment status, education, parents' place of birth, and income. See SM Section 2.1.1 for the pre-registered definitions of each control and outcome variable.
* $p < 0.10$, ** $p < 0.05$, *** $p < 0.01$

2.4 Further robustness checks

2.4.1 Accounting for rollout dynamics and different sets of controls

This section presents further robustness checks. We show that our results are robust to including different sets of controls, including different ways to control for age and region and dropping groups of pre-registered controls. We then show that our results are equivalent when we use logistic regressions rather than the pre-registered ordinary least squares (OLS). While our sample is largely representative of the Swedish population in terms of age, income, and region, we have a slight misrepresentation in terms of gender, education, and immigration status. We show that the results remain equivalent when we perform our main analysis reweighting the sample to make it representative of the Swedish population. Finally, we show that our results remain consistent when we use different inclusion criteria.

Table S10: Treatment effects controlling for each age-region interaction. To account for Sweden's vaccination rollout, our analysis controls for age-specific effects (age fixed effects), region-specific effects (region fixed effects), and the interaction of the two (region x age fixed effects), as pre-registered. We do so by using 5-year age bands. In practice, however, the Swedish rollout was more complex, and some regions used year bands smaller than 5 years. This table shows that our results are robust when we instead include 1-year bands for the age fixed effects (or 1 fixed effect for each specific age), region-specific effects, and the interaction of the two. This specification accounts for all the possible rollout strategies that each region used. We find that the effects on both vaccination uptake and intentions are very similar to those in the main specifications.

Table S10: Treatment effects controlling for each specific age interacted with region

Dependent Variable	Vaccination Uptake				Vaccination Intentions			
	(1)	(2)	(3)	(4)	(5)	(6)	(7)	(8)
Incentives Cond.	4.36***	4.36***	4.23***	4.24***	4.17***	4.16***	4.24***	4.23***
	(1.59)	(1.59)	(1.56)	(1.56)	(1.25)	(1.25)	(1.24)	(1.24)
All Nudges	1.62		0.81		2.32**		1.69*	
	(1.20)		(1.18)		(0.97)		(0.95)	
Social Impact Cond.		1.98		1.19		2.62**		2.04
		(1.63)		(1.61)		(1.28)		(1.26)
Argument Cond.		1.91		1.02		3.51***		2.78**
		(1.65)		(1.61)		(1.30)		(1.27)
Information Cond.		0.97		0.22		0.82		0.23
		(1.68)		(1.65)		(1.38)		(1.35)
No reminders Cond.	-0.77	-0.77	-1.33	-1.32	0.49	0.50	0.02	0.02
	(1.67)	(1.67)	(1.64)	(1.64)	(1.35)	(1.35)	(1.32)	(1.32)
Each Age x Region FE	yes	yes	yes	yes	yes	yes	yes	yes
Controls			yes	yes			yes	yes
Observations	8,286	8,286	8,286	8,286	8,286	8,286	8,286	8,286

Note: The table shows coefficient estimates from linear regressions of the outcome variable on each of the treatment conditions. All nudges pool the *social impact*, *argument*, and *information condition*. Heteroscedasticity robust standard errors are shown in parentheses. All regressions use the pre-registered controls: gender, fixed effects for each specific age, region, interactions between age and region, being in an at-risk group for COVID-19, civil status, having children in the household, employment status, education, parents' place of birth, and income. See SM Section 2.1.1 for the pre-registered definitions of each control and outcome variable. * $p < 0.10$, ** $p < 0.05$, *** $p < 0.01$

Tables S11 and S12: Treatment effects controlling for the timing of the survey. We fielded the survey in three waves over 6 weeks, meaning that participants answered it at different points of the rollout. The next two tables account for the timing of the survey completion by including week fixed effects. It further shows the results when interacting week fixed effects with participant's age. Once again, the results are similar and consistent with those in the main text.

Table S11: Treatment effects on incentives and all nudges controlling for the timing of the survey

Dependent Variable	Vaccination Uptake						Vaccination Intentions					
	(1)	(2)	(3)	(4)	(5)	(6)	(7)	(8)	(9)	(10)	(11)	(12)
Incentives Cond.	3.97***	3.92***	4.18***	4.36***	4.30***	4.08***	3.97***	3.92***	4.18***	4.36***	4.30***	4.08***
	(1.51)	(1.50)	(1.50)	(1.50)	(1.51)	(1.51)	(1.51)	(1.50)	(1.50)	(1.50)	(1.51)	(1.51)
All Nudges	1.25	1.20	1.17	1.21	0.89	1.14	1.25	1.20	1.17	1.21	0.89	1.14
	(1.13)	(1.13)	(1.14)	(1.13)	(1.14)	(1.14)	(1.13)	(1.13)	(1.14)	(1.13)	(1.14)	(1.14)
No reminders Cond.	-1.46	-1.31	-0.95	-0.93	-1.11	-0.91	-1.46	-1.31	-0.95	-0.93	-1.11	-0.91
	(1.58)	(1.57)	(1.58)	(1.58)	(1.59)	(1.59)	(1.58)	(1.57)	(1.58)	(1.58)	(1.59)	(1.59)
Controls	yes	yes	yes	yes	yes	yes	yes	yes	yes	yes	yes	yes
Age FE		yes						yes				
Age x Region FE			yes	yes	yes	yes			yes	yes	yes	yes
Week FE				yes						yes		
Week x Region FE					yes						yes	
Age x Week FE						yes						yes
Observations	8,286	8,286	8,286	8,286	8,286	8,286	8,286	8,286	8,286	8,286	8,286	8,286

Note: The table shows coefficient estimates from linear regressions of the outcome variable on each of the treatment conditions. All nudges pool the *social impact*, *argument*, and *information condition*. Heteroscedasticity robust standard errors are shown in parentheses. Regressions use the pre-registered controls: gender, age, region, interactions between age and region, being in an at-risk group for COVID-19, civil status, having children in the household, employment status, education, parents' place of birth, and income. See SM Section 2.1.1 for the pre-registered definitions of each control and outcome variable. * $p < 0.10$, ** $p < 0.05$, *** $p < 0.01$

Table S12: Treatment effects on all conditions controlling for the timing of the survey

Dependent Variable	Vaccination Uptake						Vaccination Intentions					
	(1)	(2)	(3)	(4)	(5)	(6)	(7)	(8)	(9)	(10)	(11)	(12)
Incentives Cond.	3.97***	3.92***	4.18***	4.36***	4.30***	4.08***	3.97***	3.92***	4.18***	4.36***	4.30***	4.08***
	(1.51)	(1.50)	(1.50)	(1.50)	(1.51)	(1.51)	(1.51)	(1.50)	(1.50)	(1.50)	(1.51)	(1.51)
Social Impact Cond.	1.37	1.44	1.42	1.43	1.21	1.16	1.37	1.44	1.42	1.43	1.21	1.16
	(1.55)	(1.54)	(1.55)	(1.54)	(1.55)	(1.56)	(1.55)	(1.54)	(1.55)	(1.54)	(1.55)	(1.56)
Argument Cond.	1.62	1.47	1.35	1.31	1.06	1.22	1.62	1.47	1.35	1.31	1.06	1.22
	(1.55)	(1.55)	(1.56)	(1.56)	(1.57)	(1.57)	(1.55)	(1.55)	(1.56)	(1.56)	(1.57)	(1.57)
Information Cond.	0.74	0.68	0.74	0.88	0.38	1.04	0.74	0.68	0.74	0.88	0.38	1.04
	(1.57)	(1.57)	(1.58)	(1.58)	(1.60)	(1.59)	(1.57)	(1.57)	(1.58)	(1.58)	(1.60)	(1.59)
No reminders Cond.	-1.46	-1.31	-0.95	-0.93	-1.11	-0.91	-1.46	-1.31	-0.95	-0.93	-1.11	-0.91
	(1.58)	(1.57)	(1.58)	(1.58)	(1.59)	(1.59)	(1.58)	(1.57)	(1.58)	(1.58)	(1.59)	(1.59)
Controls	yes	yes	yes	yes	yes	yes	yes	yes	yes	yes	yes	yes
Age FE		yes						yes				
Age x Region FE			yes	yes	yes	yes			yes	yes	yes	yes
Week FE				yes						yes		
Week x Region FE					yes						yes	
Age x Week FE						yes						yes
Observations	8,286	8,286	8,286	8,286	8,286	8,286	8,286	8,286	8,286	8,286	8,286	8,286

Note: The table shows coefficient estimates from linear regressions of the outcome variable on each of the experimental conditions. Heteroscedasticity robust standard errors are shown in parentheses. Regressions use the pre-registered controls: gender, age, region, interactions between age and region, being in an at-risk group for COVID-19, civil status, having children in the household, employment status, education, parents' place of birth, and income. See SM Section 2.1.1 for the pre-registered definitions of each control and outcome variable. * $p < 0.10$, ** $p < 0.05$, *** $p < 0.01$

Tables S13 and S14: Treatment effects including different sets of controls. We pre-registered that for our main analysis we would control for gender, age, region, interactions between age and region, being in an at-risk group for COVID-19, civil status, having children in the household, employment status, education, parents' place of birth, and income. The next two tables show that our results are robust to including different sets of control variables.

Table S13: Treatment effects on incentives and all nudges including different sets of controls

Dependent Variable	Vaccination Uptake						Vaccination Intentions					
	(1)	(2)	(3)	(4)	(5)	(6)	(7)	(8)	(9)	(10)	(11)	(12)
Incentives Cond.	4.03***	4.33***	4.32***	4.14***	4.08***	4.18***	3.94***	3.69***	3.74***	3.68***	3.64***	3.72***
	(1.54)	(1.53)	(1.53)	(1.50)	(1.50)	(1.50)	(1.22)	(1.22)	(1.22)	(1.20)	(1.20)	(1.20)
All Nudges	2.15*	2.02*	2.02*	1.28	1.26	1.17	2.74***	2.48***	2.49***	1.92**	1.84**	1.77*
	(1.15)	(1.16)	(1.16)	(1.14)	(1.14)	(1.14)	(0.94)	(0.94)	(0.94)	(0.93)	(0.93)	(0.93)
No reminders Cond.	-0.87	-0.38	-0.36	-1.10	-1.08	-0.95	0.71	0.74	0.69	0.13	0.11	0.21
	(1.61)	(1.61)	(1.61)	(1.58)	(1.58)	(1.58)	(1.31)	(1.32)	(1.32)	(1.30)	(1.29)	(1.29)
Age x Region FE		yes	yes	yes	yes	yes		yes	yes	yes	yes	yes
Gender			yes	yes	yes	yes			yes	yes	yes	yes
Socioeconomic Status				yes	yes	yes				yes	yes	yes
Family					yes	yes					yes	yes
COVID-19 Risk-group						yes						yes
Observations	8,286	8,286	8,286	8,286	8,286	8,286	8,286	8,286	8,286	8,286	8,286	8,286

Note: The table shows coefficient estimates from linear regressions of the outcome variable on each of the treatment conditions. All nudges pool the *social impact*, *argument*, and *information condition*. Heteroscedasticity robust standard errors are shown in parentheses. See SM Section 2.1.1 for the pre-registered definitions of each control and outcome variable.

* $p < 0.10$, ** $p < 0.05$, *** $p < 0.01$

Table S14: Treatment effects on all conditions including different sets of controls

Dependent Variable	Vaccination Uptake						Vaccination Intentions					
	(1)	(2)	(3)	(4)	(5)	(6)	(7)	(8)	(9)	(10)	(11)	(12)
Incentives Cond.	4.03***	4.33***	4.32***	4.14***	4.08***	4.18***	3.94***	3.68***	3.74***	3.67***	3.64***	3.71***
	(1.54)	(1.53)	(1.53)	(1.50)	(1.50)	(1.50)	(1.22)	(1.22)	(1.22)	(1.20)	(1.21)	(1.20)
Social Impact Cond.	2.23	2.29	2.28	1.60	1.59	1.42	3.20**	2.94**	2.98**	2.46**	2.35*	2.22*
	(1.57)	(1.57)	(1.57)	(1.55)	(1.55)	(1.55)	(1.25)	(1.26)	(1.26)	(1.24)	(1.24)	(1.23)
Argument Cond.	2.60	2.19	2.20	1.35	1.32	1.35	3.84***	3.42***	3.40***	2.75**	2.70**	2.72**
	(1.59)	(1.59)	(1.59)	(1.56)	(1.56)	(1.56)	(1.25)	(1.26)	(1.26)	(1.24)	(1.24)	(1.24)
Information Cond.	1.63	1.58	1.58	0.90	0.87	0.74	1.17	1.06	1.07	0.52	0.45	0.35
	(1.60)	(1.61)	(1.61)	(1.59)	(1.59)	(1.58)	(1.32)	(1.33)	(1.33)	(1.31)	(1.30)	(1.30)
No reminders Cond.	-0.87	-0.38	-0.36	-1.10	-1.08	-0.95	0.71	0.74	0.69	0.13	0.11	0.21
	(1.61)	(1.61)	(1.61)	(1.58)	(1.58)	(1.58)	(1.31)	(1.32)	(1.32)	(1.30)	(1.29)	(1.29)
Age x Region FE		yes	yes	yes	yes	yes		yes	yes	yes	yes	yes
Gender			yes	yes	yes	yes			yes	yes	yes	yes
Socioeconomic Status				yes	yes	yes				yes	yes	yes
Family					yes	yes					yes	yes
COVID-19 Risk-group						yes						yes
Observations	8,286	8,286	8,286	8,286	8,286	8,286	8,286	8,286	8,286	8,286	8,286	8,286

Note: The table shows coefficient estimates from linear regressions of the outcome variable on each of the treatment conditions. Heteroscedasticity robust standard errors are shown in parentheses. See SM Section 2.1.1 for the pre-registered definitions of each control and outcome variable.
* $p < 0.10$, ** $p < 0.05$, *** $p < 0.01$

2.4.2 Logit regressions

Table S15: Treatment effects using logit regressions. We pre-registered that we would use ordinary least squares (OLS) regressions for our analysis. This table shows that the effects remains robust when we instead used logit regressions.

Table S15: Treatment effects using logit regressions

Dependent Variable	Vaccination Uptake		Vaccination Intentions	
	(1)	(2)	(3)	(4)
Incentives Cond.	0.23***	0.23***	0.32***	0.32***
	(0.09)	(0.09)	(0.11)	(0.11)
All Nudges	0.06		0.15*	
	(0.06)		(0.08)	
Social Impact Cond.		0.08		0.20*
		(0.08)		(0.11)
Argument Cond.		0.08		0.23**
		(0.09)		(0.11)
Information Cond.		0.04		0.02
		(0.09)		(0.10)
No reminders Cond.	-0.05	-0.05	0.01	0.01
	(0.08)	(0.08)	(0.10)	(0.10)
Age x Region FE	yes	yes	yes	yes
Controls	yes	yes	yes	yes
Observations	8,243	8,243	8,194	8,194

Note: The table shows coefficient estimates from logit regressions with heteroscedasiticity-robust standard errors of the outcome variable on each of the treatment conditions. All nudges pool the *social impact*, *argument*, and *information condition*. Heteroscedasticity robust standard errors are shown in parentheses. Regressions use the pre-registered controls: gender, age, region, interactions between age and region, being in an at-risk group for COVID-19, civil status, having children in the household, employment status, education, parents' place of birth, and income. See SM Section 2.1.1 for the pre-registered definitions of each control and outcome variable. Some observations are dropped during estimation because the region and age interactions predict the vaccination uptake perfectly in smaller regions.
* $p < 0.10$, ** $p < 0.05$, *** $p < 0.01$

2.4.3 Sample weights

Table S16: Treatment effects with sample weights. Our sample is representative of the Swedish population (aged 18-49) in terms of age, region, and income. However, we have a slight overrepresentation of people with college education, an underrepresentation of people whose parents or themselves were born abroad, and a slight overrepresentation of women (as discussed in SM Section 2.1.2).

In this table, we show that results do not change when using sampling weights for adjustment. We replicate our main analysis (see SM Section 2.3.1), but reweight individuals with college education, immigration background, and women in such a way that the estimates are based on a sample which has the same characteristics as the general population in Sweden (using raking adjustments to survey sampling weights).

Table S16: Treatment effects with sample weights

Dependent Variable	Vaccination Uptake		Vaccination Intentions	
	(1)	(2)	(3)	(4)
Incentives Cond.	3.92**	3.92**	4.05***	4.05***
	(1.77)	(1.77)	(1.45)	(1.45)
All Nudges	1.59		1.98*	
	(1.32)		(1.10)	
Social Impact Cond.		2.09		2.03
		(1.76)		(1.48)
Argument Cond.		1.15		2.85*
		(1.83)		(1.49)
Information Cond.		1.52		1.01
		(1.84)		(1.53)
No Reminders Cond.	-2.02	-2.02	-0.54	-0.54
	(1.85)	(1.85)	(1.60)	(1.60)
Age x Region FE	yes	yes	yes	yes
Controls	yes	yes	yes	yes
Observations	8,286	8,286	8,286	8,286

Note: The table shows coefficient estimates from linear regressions of the outcome variable on each of the treatment conditions. All nudges pool the *social impact*, *argument*, and *information condition*. Heteroscedasticity robust standard errors are shown in parentheses. Regressions use the pre-registered controls: gender, age, region, interactions between age and region, being in an at-risk group for COVID-19, civil status, having children in the household, employment status, education, parents' place of birth, and income. See SM Section 2.1.1 for the pre-registered definitions of each control and outcome variable. We used raking adjustmented survey weights, which we constructed using the variable definitions according to Statistics Sweden and as discussed in SM Section 2.2.3.
* $p < 0.10$, ** $p < 0.05$, *** $p < 0.01$

2.4.4 Different inclusion criteria

Table S17: Treatment effects including participants who did not finish the survey. In the main specification we exclude observations from all participants who did not finish the survey (see Methods and Materials in SM Section 1 for a discussion about the inclusion of the participants). In this table, we show that including all participants who went through the experimental intervention but did not finish the rest of the questionnaire does not affect our results.

Table S17: Treatment effects including participants who did not finish the survey

Dependent Variable	Vaccination Uptake	
	(1)	(2)
Incentives Cond.	3.93***	3.94***
	(1.51)	(1.51)
All Nudges	0.36	
	(1.13)	
Social Impact Cond.		0.88
		(1.55)
Argument Cond.		0.29
		(1.55)
Information Cond.		-0.09
		(1.58)
No reminders Cond.	-0.87	-0.87
	(1.58)	(1.58)
Age x Region FE	yes	yes
Controls	yes	yes
Observations	8,397	8,397

Note: The table shows coefficient estimates from linear regressions of the outcome variable on each of the treatment conditions. All nudges pool the *social impact*, *argument*, and *information condition*. Heteroscedasticity robust standard errors are shown in parentheses. Regressions use the pre-registered controls: gender, age, region, interactions between age and region, being in an at-risk group for COVID-19, civil status, having children in the household, employment status, education, parents' place of birth, and income. See SM Section 2.1.1 for the pre-registered definitions of each control and outcome variable.
* $p < 0.10$, ** $p < 0.05$, *** $p < 0.01$

Table S18: Treatment effects including participants who were already vaccinated. In the main specification, we exclude observations from all participants who were already vaccinated (see Methods and Materials in SM Section 1 for a discussion about the inclusion of the participants). In this table, we show that including all participants who that already received the vaccine before completing the survey does not affect our results.

Table S18: Treatment effects including participants who were already vaccinated

Dependent Variable	Vaccination Uptake		Vaccination Intentions	
	(1)	(2)	(3)	(4)
Incentives Cond.	4.08***	4.08***	3.43***	3.43***
	(1.47)	(1.47)	(1.18)	(1.18)
All Nudges	1.26		1.85**	
	(1.11)		(0.91)	
Social Impact Cond.		1.50		2.28*
		(1.51)		(1.21)
Argument Cond.		1.62		2.92**
		(1.52)		(1.20)
Information Cond.		0.66		0.32
		(1.56)		(1.28)
No reminders Cond.	-0.77	-0.77	0.37	0.37
	(1.55)	(1.55)	(1.26)	(1.26)
Age x Region FE	yes	yes	yes	yes
Controls	yes	yes	yes	yes
Observations	8,510	8,510	8,510	8,510

Note: The table shows coefficient estimates from linear regressions of the outcome variable on each of the treatment conditions. All nudges pool the *social impact*, *argument*, and *information condition*. Heteroscedasticity robust standard errors are shown in parentheses. Regressions use the pre-registered controls: gender, age, region, interactions between age and region, being in an at-risk group for COVID-19, civil status, having children in the household, employment status, education, parents' place of birth, and income. See SM Section 2.1.1 for the pre-registered definitions of each control and outcome variable.
* $p < 0.10$, ** $p < 0.05$, *** $p < 0.01$

Table S19: Treatment effects including participants who participated more than once. In the main specification, we exclude observations from all participants who filled out the survey more than once and got exposed to different treatment conditions (see Methods and Materials in SM Section 1 for a discussion about the inclusion of the participants). In this table, we show that including all participants who participated more than once does not affect our results.

Table S19: Treatment effects including participants who participated more than once

Dependent Variable	Vaccination Uptake		Vaccination Intentions	
	(1)	(2)	(3)	(4)
Incentives Cond.	3.70**	3.70**	3.61***	3.61***
	(1.44)	(1.44)	(1.14)	(1.14)
All Nudges	0.80		1.21	
	(1.09)		(0.89)	
Social Impact Cond.		1.07		1.96
		(1.50)		(1.19)
Argument Cond.		0.73		1.91
		(1.50)		(1.20)
Information Cond.		0.58		-0.27
		(1.54)		(1.25)
No Reminders Cond.	-0.52	-0.52	-0.13	-0.12
	(1.52)	(1.52)	(1.23)	(1.23)
Age x Region FE	yes	yes	yes	yes
Controls	yes	yes	yes	yes
Observations	8,923	8,923	8,923	8,923

Note: The table shows coefficient estimates from linear regressions of the outcome variable on each of the treatment conditions. All nudges pool the *social impact*, *argument*, and *information condition*. Heteroscedasticity robust standard errors are shown in parentheses. Regressions use the pre-registered controls: gender, age, region, interactions between age and region, being in an at-risk group for COVID-19, civil status, having children in the household, employment status, education, parents' place of birth, and income. See SM Section 2.1.1 for the pre-registered definitions of each control and outcome variable.
* $p < 0.10$, ** $p < 0.05$, *** $p < 0.01$

Table S20: Treatment effects including participants who could not be linked to administrative vaccination records. In the main specification, we exclude observations from all participants for whom we could not match the data with their vaccination records, likely because of a typo in their social security number (see Methods and Materials in SM Section 1 for a discussion about the inclusion of the participants). In this table, we provide the results for intentions and appointment clicks including the participants who could not be linked to the administrative vaccination records. While we cannot know whether they indeed vaccinated, we see that treatment effects on the other outcome variables are similar to those for the whole sample.

Table S20: Treatment effects including participants who could not be linked

Dependent Variable	Intention Measure: Main Yes=100 or No=0		Continuous 0 to 100%		Time 1 to 10		Appointment Link Click Yes=100 or No=0	
	(1)	(2)	(3)	(4)	(5)	(6)	(7)	(8)
Incentives Cond.	3.47***	3.47***	2.08**	2.07**	-0.17**	-0.17**	4.75***	4.75***
	(1.18)	(1.18)	(0.93)	(0.93)	(0.08)	(0.08)	(0.98)	(0.98)
All Nudges	1.79**		1.16		-0.05		-0.03	
	(0.91)		(0.73)		(0.06)		(0.58)	
Social Impact Cond.		2.26*		0.56		-0.02		0.94
		(1.21)		(0.99)		(0.08)		(0.84)
Argument Cond.		2.89**		2.05**		-0.10		0.19
		(1.20)		(0.99)		(0.08)		(0.82)
Information Cond.		0.19		0.89		-0.05		-1.28*
		(1.28)		(1.04)		(0.09)		(0.75)
No reminders Cond.	0.37	0.37	-1.88*	-1.88*	0.09	0.09		
	(1.26)	(1.26)	(1.03)	(1.03)	(0.08)	(0.08)		
Age x Region FE	yes	yes	yes	yes	yes	yes	yes	yes
Controls	yes	yes	yes	yes	yes	yes	yes	yes
Observations	8,515	8,515	8,488	8,488	8,474	8,474	7,374	7,374

Note: The table shows coefficient estimates from linear regressions of the outcome variable on each of the treatment conditions. All nudges pool the *social impact*, *argument*, and *information condition*. Heteroscedasticity robust standard errors are shown in parentheses. Regressions use the pre-registered controls: gender, age, region, interactions between age and region, being in an at-risk group for COVID-19, civil status, having children in the household, employment status, education, parents' place of birth, and income. See SM Section 2.1.1 for the pre-registered definitions of each control and outcome variable. In columns (7) and (8) we do not observe people in no reminders condition, who did not get an appointment link.
* $p < 0.10$, ** $p < 0.05$, *** $p < 0.01$

2.4.5 Multiple hypothesis testing for vaccination uptake

Our RCT tests the effects of five treatment arms: incentives condition, social impact condition, argument condition, information condition, and no-reminders condition.

In the main text of paper, we report tests of the null hypothesis that each condition has an effect on vaccination uptake. If, instead, the reader is interested in testing the null hypothesis that no intervention affects vaccination uptake, one needs to account for multiple hypothesis testing, which can be done through adjustments to p-values.

To study whether we can reject the null that none of the interventions affects vaccination uptake, we used the Bonferroni adjustment, which is the most conservative of the standard p-value adjustments for multiple testing. This method implies that we can reject the null hypothesis if the p-value of a t-test for significance is smaller than 0.05 divided by the number of tests.

In our pre-analysis plan, we pre-registered that for our main analysis we would compare the incentives condition, social impact condition, argument condition and information condition with the control condition. We pre-registered that the comparison between the no-reminders condition is a secondary analysis. Hence, we need to account for four tests.

Accounting for these four tests implies that we can reject the null whenever $p < 0.0125$. Since the main effect of incentives on vaccination uptake yields a p-value of $p = 0.005$ (see Section 2.3), we conclude that our result is robust to adjustments for multiple hypothesis testing.

2.5 Treatment effects based on participants' characteristics

As secondary analyses, we pre-registered that we would study whether there are heterogeneities in treatment effects for people with different economic preferences, personality traits, vaccine beliefs, and vaccine knowledge. We provide the results for the pre-registered analysis in this section.

Specification: We use a fully interacted OLS model where we prespecify all simple interactions of the interventions with each of the different individual characteristics (we will call these different characteristics "$measure_i$"). That is, we regress Vaccination uptake on a set of experimental condition dummies and the interaction between treatment dummies and individual characteristics:

$VaccinationUptake_i = b_0 + b_1 * 1(Incentives)_i + b_2 * 1(Arguments)_i + b_3 * 1(Information)_i + b_4 * 1(Socialimpact)_i + b_5 * 1(Noreminders)_i + b_6 * 1(Incentives)_i * measure_i + b_7 * 1(Arguments)_i * measure_i + b_8 * 1(Information)_i * measure_i + b_9 * 1(Socialimpact)_i * measure_i + b_{10} * 1(Noreminders)_i * measure_i + b_{11} * measure_i + b_{12} * X_i + e_i$

where $1(t)_i$ has a value of 1 if participant i is in the treatment condition t and a value of 0 otherwise, X_i is a vector of the pre-registered control variables (consisting of $measure_i$, gender dummies, age dummies, region dummies, interactions between age and region, being in an at-risk group for COVID-19, civil status dummies, a dummy for children in the household, dummies for employment status, dummies for education, dummies for parents' place of birth, and income), and e_i is an individual specific error robust to heteroscedasticity. For interactions with sociodemographics, we only include region fixed effects.

We provide results (b_6 - b_{10} and baseline correlation, b_{11}) for all individual characteristics.

Summary of results: We do not find robust heterogeneities. Rather, incentives increase vaccination rates similarly for all subgroups.

As an illustration, Figure S8 below shows the results for median splits across sociodemographic variables. As Figure S8 indicates, the coefficient sizes barely vary across age groups, gender, education status, or socioeconomic status.

Tables S21 and S22 give the more detailed results of interacting an array of variables with each treatment. The interactions which are statistically significant at the 5% level are: people with higher income tend to have lower vaccination uptake in the information treatment; people who are more willing to take risks are more likely to react to the incentives and the argument conditions;

and the small share of people who has tested positive for COVID-19 in the past is more likely to react to incentives.[4]

However, note that we examine 40 interactions for sociodemographics and 65 interactions with economic preferences, personality, and COVID-19 related variables. For a total of 105 interactions, we only find 4 statistically significant interactions at the 5%-level and none at the 1% level. This is not more than is expected to occur by chance. Accordingly, there are no systematic heterogeneous impacts on vaccination uptake based on the variables we measure. We therefore refrain from drawing any conclusions from the few statistically significant interactions.

[4]One reason for imprecise estimates for people with immigration background may be that we have relatively few observations in the sample.

2.5.1 Heterogeneities based on sociodemographics

Figure S8: Treatment effects of incentives across sociodemographic groups. This figure represents the treatment effects for all the sample, and for each of different partitions based on sociodemographic factors. The figure shows that incentives increase vaccination uptake similarly among all the subgroups according to median splits of the data.

Figure S8: Treatment effects of incentives across sociodemographic groups

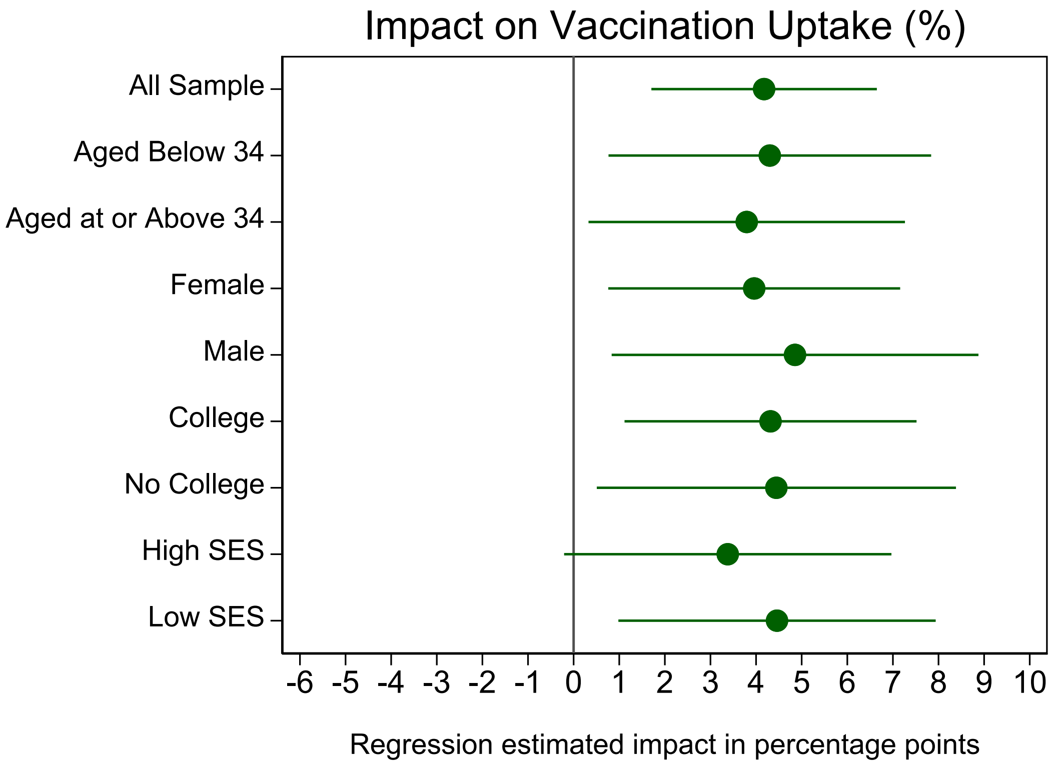

Note: Participants who have at least one parent with a non-European background, who are unemployed, or who have below median income are categorized as Low SES. The rest of the participants are categorized as high SES.

Table S21: Heterogeneous treatment effects based on sociodemographics. Using the specification described in the introduction of this section, this table reports the heterogeneous treatment effects for each condition based on participants' sociodemographics. The table first displays the correlation between the variable and vaccination uptake, showing strong correlations with college education, income, immigration background, and employment status. However, there are no relevant treatment effect heterogeneities with any of the variables nor conditions.

Table S21: Heterogeneous treatment effects based on sociodemographics

Measure:	Interaction effect with treatment					
	Baseline Corr.	Incent. Cond.	Social Imp. Cond.	Argument Cond.	Info. Cond.	No reminders Cond.
Age	0.02	-0.07	0.05	-0.20	-0.12	-0.21
	(0.10)	(0.18)	(0.18)	(0.19)	(0.19)	(0.20)
Female	0.28	-0.33	-2.21	-0.86	3.00	1.13
	(1.73)	(3.11)	(3.18)	(3.20)	(3.25)	(3.25)
Single	-0.92	2.60	3.22	-5.78	-0.19	-1.04
	(1.95)	(3.44)	(3.60)	(3.71)	(3.68)	(3.73)
Has children	-0.10	-1.94	-2.19	-4.13	-3.99	-1.61
	(1.71)	(3.06)	(3.14)	(3.16)	(3.19)	(3.22)
College	10.92***	-0.64	-1.67	-0.55	1.55	0.70
	(1.71)	(3.07)	(3.16)	(3.22)	(3.21)	(3.25)
Income	3.24***	0.00	-0.00	0.00	-0.00**	-0.00
	(0.80)	(0.00)	(0.00)	(0.00)	(0.00)	(0.00)
Foreign	-13.32***	-1.83	2.38	-9.74	-7.86	5.05
	(4.43)	(8.71)	(9.13)	(8.74)	(9.18)	(8.94)
Unemployed	-16.56***	3.30	-0.33	-8.05	2.13	4.65
	(5.00)	(8.61)	(9.43)	(10.39)	(9.29)	(10.52)
Region FE	yes	yes	yes	yes	yes	yes

Note: The table shows coefficient estimates from linear regressions of the outcome variable on each of the treatment conditions. Heteroscedasticity robust standard errors are shown in parentheses. Regressions control for region fixed effects. See SM Section 2.1.1 for the pre-registered definitions of each control and outcome variable.
* $p < 0.10$, ** $p < 0.05$, *** $p < 0.01$

2.5.2 Heterogeneities based on economic preferences, personality traits, and COVID-19 related variables

Table S22: Heterogeneous treatment effects based on economic preferences, personality traits, and COVID-19 related variables. Using the specification described in the introduction of this section, this table reports the heterogeneous treatment effects for each condition based on participants' economic preferences, personality traits, and several COVID-19 specific variables. The table first displays the correlation between the variable and vaccination uptake, showing strong correlations with most variables. Yet, there largely do not seem to exist any clear treatment effect heterogeneities with any of the variables nor conditions.

Table S22: Heterogeneous treatment effects based econ. pref, personality, and COVID-19 variables

Measure:	Interaction effect with treatment					
	Baseline Corr.	Incent. Cond.	Social Imp. Cond.	Argument Cond.	Info. Cond.	No reminders Cond.
Altruism	0.67*	0.70	0.44	0.37	-0.46	0.42
	(0.38)	(0.66)	(0.67)	(0.70)	(0.67)	(0.69)
Risk-affinity	-0.99**	1.76**	-0.48	1.93**	0.77	0.70
	(0.45)	(0.82)	(0.82)	(0.84)	(0.83)	(0.83)
Patience	-0.04	0.79	1.65*	1.80*	0.39	0.11
	(0.49)	(0.88)	(0.91)	(0.94)	(0.94)	(0.87)
Reciprocity	-1.05*	1.69	1.27	1.02	1.46	0.74
	(0.62)	(1.08)	(1.19)	(1.22)	(1.19)	(1.14)
Trust	1.53***	0.37	-0.10	-0.93	-0.31	0.00
	(0.39)	(0.73)	(0.70)	(0.72)	(0.72)	(0.76)
Procrastination	0.11	0.37	0.74	0.06	-0.25	0.86
	(0.33)	(0.57)	(0.61)	(0.59)	(0.61)	(0.62)
Norm-following	1.27***	0.59	-0.12	1.22	0.17	0.63
	(0.40)	(0.72)	(0.73)	(0.74)	(0.73)	(0.76)
COVID-19 vaccines are safe	15.81***	-1.36	-0.38	1.10	-1.55	0.28
	(0.75)	(1.41)	(1.48)	(1.45)	(1.45)	(1.41)
Vaccines cause disease	-7.98***	2.19	-1.51	-0.18	-0.25	-0.93
	(0.80)	(1.45)	(1.50)	(1.55)	(1.51)	(1.47)
Worries side effects COVID-19 vaccines	-10.00***	1.02	0.23	0.03	0.87	-0.77
	(0.61)	(1.11)	(1.13)	(1.14)	(1.14)	(1.13)
Afraid of needles	-0.98*	0.41	1.74	0.28	1.98*	-0.64
	(0.59)	(1.07)	(1.07)	(1.09)	(1.11)	(1.14)
Ever tested positive for COVID-19	-7.78***	8.40**	-3.38	1.30	0.24	-0.01
	(2.13)	(3.62)	(3.92)	(3.98)	(3.92)	(3.89)
COVID-19 risk group	-9.87***	5.35	-4.77	-0.18	-1.28	-0.24
	(3.10)	(5.30)	(6.37)	(5.93)	(6.12)	(5.54)
Age x Region FE	yes	yes	yes	yes	yes	yes
Controls	yes	yes	yes	yes	yes	yes

Note: The table shows coefficient estimates from linear regressions of the outcome variable on each of the treatment conditions. Heteroscedasticity robust standard errors are shown in parentheses. Regressions use the pre-registered controls: gender, age, region, interactions between age and region, being in an at-risk group for COVID-19, civil status, having children in the household, employment status, education, parents' place of birth, and income. See SM Section 2.1.1 for the pre-registered definitions of each control and outcome variable. * $p < 0.10$, ** $p < 0.05$, *** $p < 0.01$

2.6 Predictors of vaccination uptake and the intention-behavior gap

A key feature of our RCT is to individually match data on COVID-19 vaccination uptake from Swedish administrative records with survey data on individual characteristics, including economic preferences, personality traits, COVID-19 perceptions, and sociodemographics. We pre-registered to only focus on the data in the Control and no-reminders condition and regress (OLS with heteroscedasticity robust standard errors and same controls as for our main analysis) Vaccination Uptake and the Intention-behavior gap on different individual characteristics. We provide these results in this section.

Summary of results: We find that participants who are more altruistic, more risk averse, less reciprocous, more trusting, and adhere more to social norms are more likely to vaccinate (see Table S23). Moreover, vaccination uptake is higher among participants who believe that COVID-19 vaccines are safe, who are less worried about side-effects of the vaccines, who have never tested positive for COVID-19, are younger, have college studies, have higher income, do not have immigration background, and are employed (see Tables S24 and S25).

Moreover, we find that the intention-behavior gap is higher among participants who do not believe that COVID-19 vaccines are safe, who are worried about side-effects of the vaccines, who have previously tested positive for COVID-19, who are in a risk group for COVID-19, who are male, have no university education, and have immigration background (see Tables S27 and S28). The intention-behavior gap is not correlated with economic preferences and personality traits (see Table S26).

2.6.1 Predictors of vaccination uptake

Table S23: Predictors of vaccination uptake: economic preferences. This table reports the correlation between vaccination uptake and the economic preferences measured in the survey. Columns (1)-(7) show the correlations using each of the preferences separately, columns (8) and (9) show them in two different groups, and column (10) shows the results using all the variables jointly in the same regression. We find that participants who are more altruistic, more risk averse, less reciprocous, more trusting, and adhere more to social norms are more likely to vaccinate.

Table S23: Predictors of vaccination uptake: economic preferences

Dependent Variable	Vaccination Uptake									
	(1)	(2)	(3)	(4)	(5)	(6)	(7)	(8)	(9)	(10)
Altruism	0.68**							0.84**		0.67*
	(0.33)							(0.34)		(0.35)
Risk-affinity		-0.83**						-0.99**		-0.92**
		(0.39)						(0.41)		(0.42)
Patience			-0.08					-0.06		0.06
			(0.42)					(0.46)		(0.46)
Reciprocity				-0.88					-1.30**	-1.41**
				(0.54)					(0.54)	(0.56)
Trust					1.43***				1.24***	1.22***
					(0.34)				(0.35)	(0.36)
Procrastination						0.30			0.17	0.14
						(0.28)			(0.28)	(0.28)
Norm-adherence							1.41***		1.21***	1.10***
							(0.34)		(0.35)	(0.35)
Age x Region FE	yes	yes	yes	yes	yes	yes	yes	yes	yes	yes
Controls	yes	yes	yes	yes	yes	yes	yes	yes	yes	yes
Observations	3,887	3,887	3,887	3,887	3,887	3,887	3,887	3,887	3,887	3,887

Note: The table shows coefficient estimates from linear regressions. Heteroscedasticity robust standard errors are shown in parentheses. Regressions use the pre-registered controls: gender, age, region, interactions between age and region, being in an at-risk group for COVID-19, civil status, having children in the household, employment status, education, parents' place of birth, and income. See SM Section 2.1.1 for the pre-registered definitions of each control and outcome variable.
* $p < 0.10$, ** $p < 0.05$, *** $p < 0.01$

Table S24: Predictors of vaccination uptake: COVID-19 vaccination beliefs. This table reports the correlation between vaccination uptake and the set of variables about COVID-19 and vaccines that we included in the survey, including beliefs such as "COVID-19 vaccines are safe" and factual questions such as "I have tested positive for COVID-19". Columns (1)-(6) show each of the correlations separately, and column (7) does so jointly. Using the estimates in Column (7), we find that vaccination uptake is higher among participants who believe that COVID-19 vaccines are safe, who are less worried about side-effects of the vaccines, and who have never tested positive for COVID-19.

Table S24: Predictors of vaccination uptake: COVID-19 vaccination beliefs

Dependent Variable	Vaccination Uptake						
	(1)	(2)	(3)	(4)	(5)	(6)	(7)
COVID-19 vaccines are safe	15.98***						12.20***
	(0.68)						(0.87)
Diseases can be triggered by vaccinations		-8.16***					-0.76
		(0.70)					(0.74)
Worried about side-effects			-10.27***				-4.57***
			(0.55)				(0.67)
Worried about needles				-1.10**			0.06
				(0.52)			(0.49)
Ever tested positive for COVID-19					-7.24***		-5.98***
					(1.83)		(1.70)
In a risk group for COVID-19						-9.78***	-3.50
						(2.68)	(2.42)
Age x Region FE	yes	yes	yes	yes	yes	yes	yes
Controls	yes	yes	yes	yes	yes	yes	yes
Observations	3,887	3,887	3,887	3,887	3,887	3,887	3,887

Note: The table shows coefficient estimates from linear regressions. Heteroscedasticity robust standard errors are shown in parentheses. Regressions use the pre-registered controls: gender, age, region, interactions between age and region, being in an at-risk group for COVID-19, civil status, having children in the household, employment status, education, parents' place of birth, and income. See SM Section 2.1.1 for the pre-registered definitions of each control and outcome variable.
* $p < 0.10$, ** $p < 0.05$, *** $p < 0.01$

Table S25: Predictors of vaccination uptake: Sociodemographics. This table reports the correlation between vaccination uptake and the sociodemographic variables included in the survey. Columns (1)-(8) show each of the correlations separately, and column (9) does so jointly. We find that vaccination uptake is higher among participants who are younger, have college studies, have higher income, do not have immigration background, and are employed.

Table S25: Predictors of vaccination uptake: sociodemographics

Dependent Variable	Vaccination Uptake								
	(1)	(2)	(3)	(4)	(5)	(6)	(7)	(8)	(9)
Age	-0.03								-0.22**
	(0.09)								(0.10)
Female		0.70							0.52
		(1.47)							(1.51)
Single			-1.24						0.51
			(1.67)						(1.76)
Has Children				-0.35					-0.31
				(1.45)					(1.70)
College Education					10.90***				9.85***
					(1.46)				(1.50)
Income						3.26***			2.75***
						(0.68)			(0.79)
Immigration Background							-11.23***		-12.96***
							(3.88)		(3.89)
Unemployed								-15.30***	-10.32**
								(4.43)	(4.54)
Region FE	yes	yes	yes	yes	yes	yes	yes	yes	yes
Observations	3,887	3,887	3,887	3,887	3,887	3,887	3,887	3,887	3,887

Note: The table shows coefficient estimates from linear regressions. Heteroscedasticity robust standard errors are shown in parentheses. Regressions use only region fixed effects as controls. See SM Section 2.1.1 for the pre-registered definitions of each control and outcome variable. Income is in SEK 10,000.
* $p < 0.10$, ** $p < 0.05$, *** $p < 0.01$

Table S26: Predictors of intention-behavior gap: economic preferences. This table reports the correlation between the intention-behavior gap and the economic preferences measured in the survey. Columns (1)-(7) show the correlations using each of the preferences separately, columns (8) and (9) show them in two different groups, and column (10) shows the results using all the variables jointly in the same regression. We find very small and non-statistically significant effects for all variables.

Table S26: Predictors of intention-behavior gap: economic preferences

Dependent Variable	Intention-Behavior Gap									
	(1)	(2)	(3)	(4)	(5)	(6)	(7)	(8)	(9)	(10)
Altruism	0.08							0.01		-0.01
	(0.31)							(0.33)		(0.34)
Risk-affinity		0.39						0.37		0.32
		(0.38)						(0.40)		(0.40)
Patience			0.20					0.08		-0.01
			(0.40)					(0.43)		(0.44)
Reciprocity				0.72					0.85	0.80
				(0.53)					(0.54)	(0.56)
Trust					-0.32				-0.29	-0.32
					(0.34)				(0.34)	(0.35)
Procrastination						-0.08			-0.04	-0.05
						(0.27)			(0.27)	(0.27)
Norm-adherence							-0.43		-0.44	-0.40
							(0.33)		(0.34)	(0.34)
Age x Region FE	yes	yes	yes	yes	yes	yes	yes	yes	yes	yes
Controls	yes	yes	yes	yes	yes	yes	yes	yes	yes	yes
Observations	3,240	3,240	3,240	3,240	3,240	3,240	3,240	3,240	3,240	3,240

Note: The table shows coefficient estimates from linear regressions. Heteroscedasticity robust standard errors are shown in parentheses. Regressions use the pre-registered controls: gender, age, region, interactions between age and region, being in an at-risk group for COVID-19, civil status, having children in the household, employment status, education, parents' place of birth, and income. See SM Section 2.1.1 for the pre-registered definitions of each control and outcome variable.
* $p < 0.10$, ** $p < 0.05$, *** $p < 0.01$

2.6.2 Predictors of the intention-behavior gap

Table S27: Predictors of the intention-behavior gap: COVID-19 related variables. This table reports the correlation between the intention-behavior gap and a set of variables about COVID-19 and vaccines that we included in the survey. Using the estimates in Column (7), we find that the intention-behavior gap is higher among participants who do not believe that COVID-19 are safe, who are worried about side-effects of the vaccines, who have previously tested positive for COVID-19, and who are in a risk group for COVID-19.

Table S27: Predictors of intention-behavior gap: COVID-19 related variables

Dependent Variable	Intention-Behavior Gap						
	(1)	(2)	(3)	(4)	(5)	(6)	(7)
COVID-19 vaccines are safe	-5.73***						-4.18***
	(0.92)						(1.03)
Diseases can be triggered by vaccinations		2.53***					0.78
		(0.71)					(0.76)
Worried about side-effects			3.46***				1.71***
			(0.59)				(0.66)
Worried about needles				0.68			0.04
				(0.50)			(0.49)
Ever tested positive for COVID-19					3.20*		3.66**
					(1.76)		(1.71)
In a risk group for COVID-19						4.80*	5.20**
						(2.65)	(2.60)
Age x Region FE	yes	yes	yes	yes	yes	yes	yes
Controls	yes	yes	yes	yes	yes	yes	yes
Observations	3,240	3,240	3,240	3,240	3,240	3,240	3,240

Note: The table shows coefficient estimates from linear regressions. Heteroscedasticity robust standard errors are shown in parentheses. Regressions use the pre-registered controls: gender, age, region, interactions between age and region, being in an at-risk group for COVID-19, civil status, having children in the household, employment status, education, parents' place of birth, and income. See SM Section 2.1.1 for the pre-registered definitions of each control and outcome variable.
* $p < 0.10$, ** $p < 0.05$, *** $p < 0.01$

Table S28: Predictors of the intention-behavior gap: sociodemographics. This table reports the correlation between the intention-behavior gap and the sociodemographic variables included in the survey. Columns (1)-(8) show each of the correlations separately, and column (9) does so jointly. We find that the intention-behavior gap is higher among participants who are male, have no university education, and have immigration background.

Table S28: Predictors of intention-behavior gap: sociodemographics

Dependent Variable	Intention-Behavior Gap								
	(1)	(2)	(3)	(4)	(5)	(6)	(7)	(8)	(9)
Age	0.05								0.13
	(0.08)								(0.10)
Female		-4.15***							-4.02***
		(1.39)							(1.44)
Single			-1.63						-2.70*
			(1.55)						(1.63)
Has Children				0.52					-0.27
				(1.36)					(1.61)
College Education					-6.46***				-5.56***
					(1.39)				(1.42)
Income						-1.23*			-1.43*
						(0.65)			(0.75)
Immigration Background							6.68*		7.76**
							(3.93)		(3.91)
Unemployed								8.23*	6.33
								(4.67)	(4.87)
Region FE	yes	yes	yes	yes	yes	yes	yes	yes	yes
Observations	3,240	3,240	3,240	3,240	3,240	3,240	3,240	3,240	3,240

Note: The table shows coefficient estimates from linear regressions. Heteroscedasticity robust standard errors are shown in parentheses. Regressions use only region fixed effects as controls. See SM Section 2.1.1 for the pre-registered definitions of each control and outcome variable. Income in SEK 10,000. * $p < 0.10$, ** $p < 0.05$, *** $p < 0.01$

2.7 Survey responses to the nudge conditions

2.7.1 Social impact condition

In the *social impact condition*, we asked participants to make a list of 4 people that would benefit if the participant would get vaccinated. The purpose of this intervention was to make participants aware of the social impact of getting vaccinated. We also provided participants with an illustration of such a list, written by one of the authors of this study, which likely reinforced the perceived social impact of vaccination.

Participants seem to have taken considerable care when filling in the list. Out of the 1,114 participants in the social impact condition, only 29 gave an input containing 2 characters or less on any of the four list items (by design, subjects could not leave the input fields empty). Most participants put parents and other close relatives on the list, for example, 780 participants mentioned "mom" or "dad" (mamma/pappa) on the list.

2.7.2 Argument condition

In the *arguments condition*, we asked participants to write down an argument that could convince another person to get vaccinated as soon as possible. We also gave participants the opportunity to share this argument with a person who did not plan to get vaccinated, which 80% of participants did. We then selected the arguments of ten participants and, if the participants agreed, shared the argument with a person on Amazon Mechanial Turk.

Most of the 1,076 participants took the task seriously and only 10% of them gave an argument with fewer than 40 characters. Common arguments included taking the vaccine to protect others, with 260 answers containing the string "others" (andra). Many also mentioned that taking the vaccine enables society to get back normal; 116 answers mentions "normal", "normally" or "normality" (normal/normalt/normalitet). Another frequent argument was to put forth that the risk of side effects from the vaccines is much smaller than the risks of COVID-19. Some appealed to a sense of duty or responsibility, with 65 participants making an argument containing the words "duty", "responsibility" or "obligation" (plikt/ansvar/skydlighet).

2.7.3 Information condition

In the information condition, we asked participants to answer the following questions on vaccine effectiveness and safety:

- What do you think, how effective are the COVID-19 vaccines that are used in Sweden for your age group in reducing deaths from COVID-19? (Response scale: 0 – 100%)
- What do you think, how many deaths in the EU and the USA have been confirmed to have a connection to the COVID-19 vaccines that are used in Sweden for your age group? (Response scale: 0 deaths, 1-5 deaths, 5-10 deaths, 11-20 deaths, 21-50 deaths, 51-100 deaths, 101-200 deaths, 201-500 deaths, 501-1'000 deaths, 1'001-2'000 deaths, 2'001-5'000 deaths, 5'001-10'000 deaths, >10'000 deaths)

We then gave them the correct answer based on the evidence available at the time of the trial which was that the Pfizer-BioNTech vaccine prevented 97% of deaths from COVID-19 and that there were no confirmed cases of deaths due to the Pfizer-BioNTech or Moderna COVID-19 vaccines neither in the US nor in the EU. In this section, we provide the distribution of participants' answers to these two questions, that is, their prior beliefs about vaccine effectiveness and safety.

Figure S9: Beliefs about COVID-19 vaccine effectiveness in preventing deaths, correct answer: 91-100%

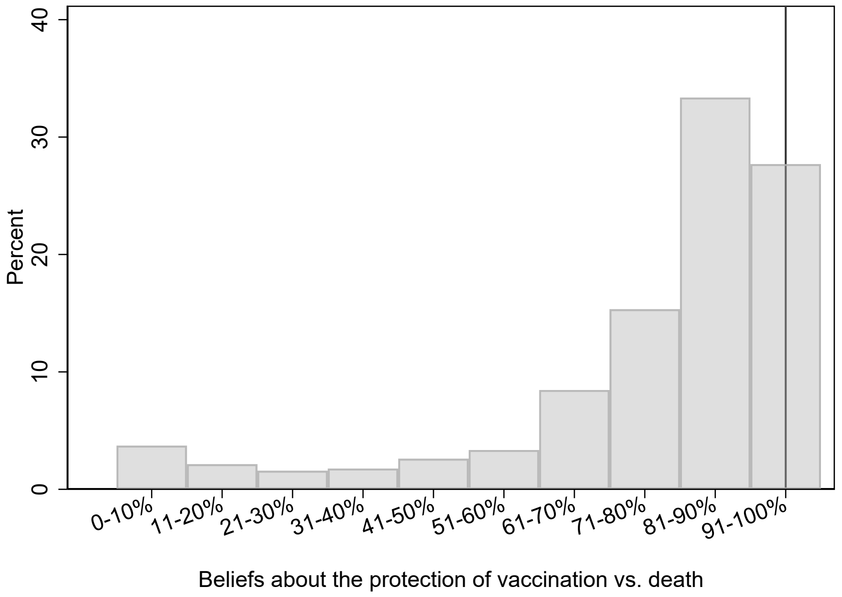

Figure S10: Beliefs about the number of deaths caused by COVID-19 vaccines (Pfizer), correct answer: 0

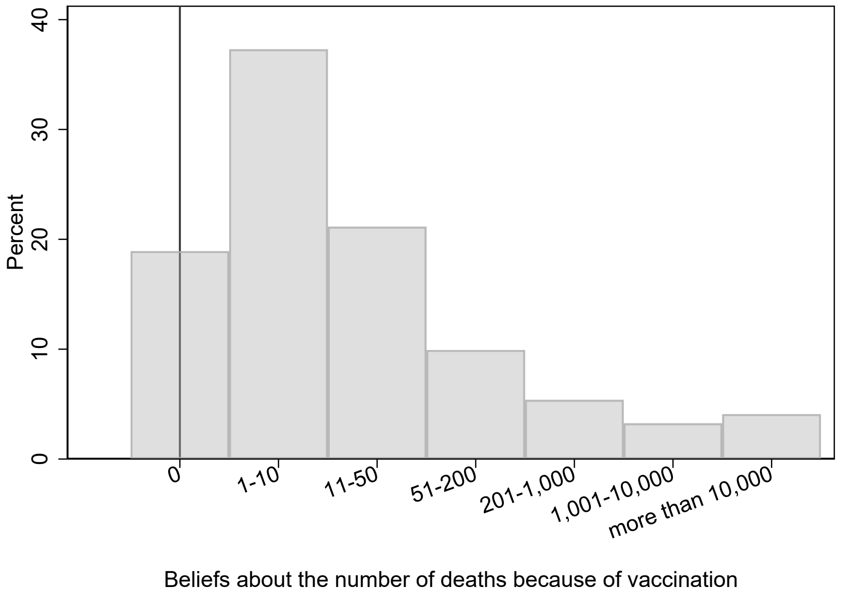

2.8 Expert predictions of treatment condition impacts on vaccination uptake and intentions vs. actual impacts

We conducted an online survey to collect expert predictions about the impact of our interventions on vaccination intention and uptake. We implemented this survey on the newly established Social Science Prediction Platform, which allows for the systematic collection and assessment of expert forecasts of the effects of untested social programs.

The data were collected during the period June 29, 2021, to August 15, 2021. The sample consisted of 53 participants (17% Professors, 13% Assistant Professors, 21% Postdocs, 28% PHD Students and 21% other researchers). The majority of respondents were Economists (81%).

Participants were told that we were currently conducting an RCT with the aim to increase COVID-19 vaccination uptake that started at the beginning of June. Their task was to predict the outcomes of the study. We explained all experimental conditions to them, told them that we would link the trial data with population-wide Swedish administrative records for vaccinations (but had not yet linked the data), and described our two main outcome measures on vaccination intentions and vaccination uptake. We then asked the experts to predict the treatment effect on the two main outcome variables for each condition. Responses were made using a slider from -15 percentage points to +15 percentage points.

Below, we show the predicted and actual difference to the control group by treatment in 1) vaccination uptake in Figure S11 and 2) vaccination intentions in Figure S12. The results have to be interpreted carefully, as we only have a convenience sample limited in size.

Interestingly, experts predicted a smaller impact of incentives on vaccination uptake than their effects were. On average, however, they predicted similar effects of nudges (predicted effects were slightly higher than actual effects) to the ones that we found.

Figure S11: Actual vs. predicted impact on vaccination uptake

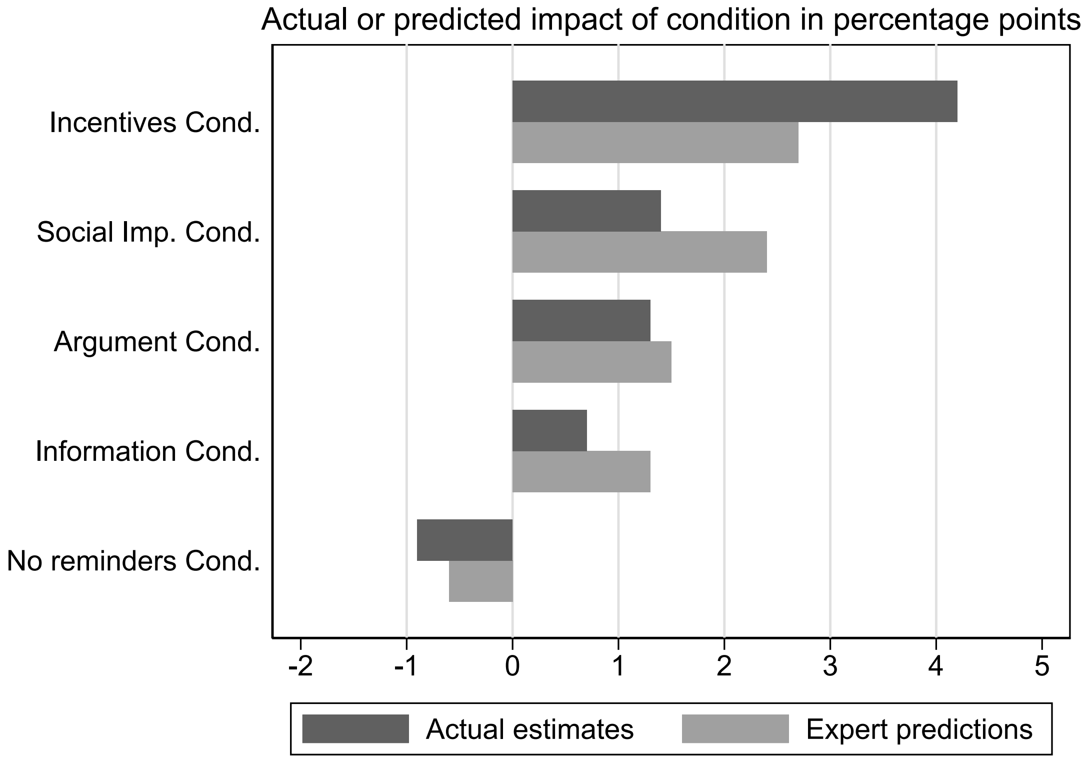

Figure S12: Actual vs. predicted impact on intentions

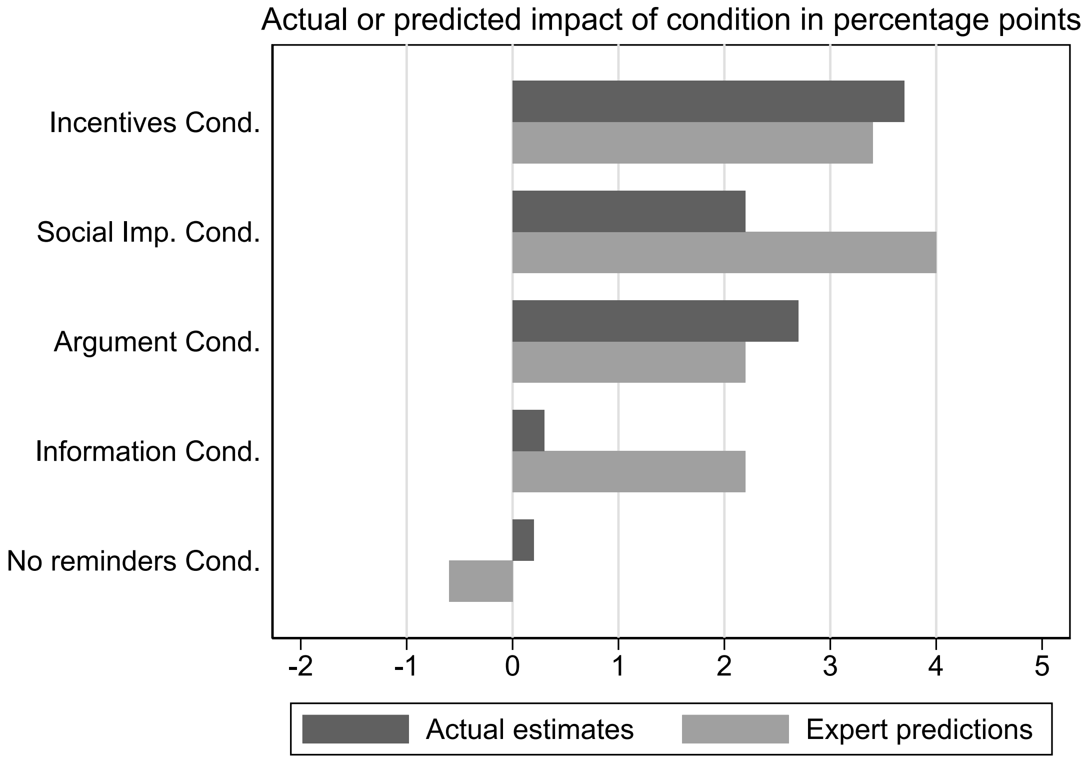

2.9 Perspectives on cost-effectiveness

A natural question is whether paying people to get vaccinated is cost effective. To answer this question, one must determine the benefits of increasing vaccination rates and compare them to the cost incurred.

At a 75% vaccination rate, offering monetary incentives to the whole Swedish adult population would cost around SEK 1.2 billion ($140 million). Using a widely applied value of a statistical life in Sweden (SEK 44 million, *42*) suggests that the program would be cost-effective if it saves at least 28 lives, or 0.035 lives per 10,000 adults.

Assessing how many lives the program would save is complex and likely to depend on a range of time and place-specific factors. However, we can get some indication from the few existing studies that estimate the impact of vaccination rates on COVID-19 mortality rates. Gupta and coauthors (*43*) measure the effect of vaccination on COVID-19 death across USA states. Until May 9, 2021, the number of saved lives ranged from 1.1 per 10,000 adults in Hawaii to 11.7 per 10,000 in New York, with 5.5 saved lives per 10 000 in the average state. The modeling in Galvani, Mohandas, and Schneider (*44*) provides comparable estimates, with 279,000 saved lives, or 8.4 saved lives per 10,000 up to the end of June 2021. These studies were carried out when between 40-50% of the adult population in the USA was fully vaccinated. Assuming that the effects of increasing vaccination rates are constant with respect to the proportion vaccinated, these results suggest that increasing vaccination rates by 1 percentage point roughly saved 0.15 lives per 10,000 adults. Hence, the direct benefits from offering monetary incentives and thereby increasing vaccination rates by 4 percentage points (0.60 lives per 10,000 adults) are likely to be higher than the costs (0.035 lives per 10 000 adults).

Besides these direct benefits of saving lives, the benefits of increasing vaccination rates also include indirect effects such as increasing the overall immunity in a country, lowering hospitalization rates and medical costs, and stimulating economic growth. Directly calculating these benefits is very difficult, and the calculation depends crucially on a large set of parameter values that will vary across countries and across different time periods of the epidemic. Hence, it is difficult to do this calculation in a way that is likely to generalize to multiple settings. Yet, if the direct benefits in terms of saved lives are higher than the costs, including these additional indirect effects does only make it more cost-effective to use incentives.

It is crucial to note that, unlike some government interventions that are intended to save lives (e.g., using more expensive but safer building materials), the $140 million is not a cost that only has

the value of preventing death. Rather, the money being spent is going to the pockets of Swedish citizens that can then be spent and enjoyed. Thus, the true cost of the program is substantially less and will depend on the cost of taxation and reallocation. In fact, many countries have been providing economic stimulus packages to their citizens that are considered cost-effective even if they do not also increase vaccination rates, such as the $1,400 per person that the USA has sent to its citizens as of September 2021.

Overall, the cost-effectiveness of the monetary incentives intervention that we document in this paper will be highly dependent on the country and timing of the intervention, along with other assumptions (e.g., the value of giving citizens money) that will have to be made by local policy-makers when considering such an intervention. However, based on the available estimates of how many lives have been saved due to vaccinations in the USA, the indirect benefits of increasing vaccination rates, and the fact that the monetary payments are not wasted but go to citizens' pockets, it seems clear that the intervention has potential to pass a cost-effectiveness test in many contexts.

2.10 Experimental instructions

What follows are the completed instructions from the trial, including all questions asked and all details on each experimental condition. The trial was conducted in Swedish, we include an English translation of the text. For the items used in previous research, whenever possible we used researcher-provided translations of the questions.

Consent Form

Background

We are a group of researchers led by Professor Erik Wengström from Lund University who are conducting a study on vaccination against the coronavirus (COVID-19). By participating in the study, you contribute to research in this area. No special knowledge is required to participate in the study, and your answers will remain confidential and anonymous.

Data management and confidentiality

The project will collect and record information about you and your opinions. Your answers will be protected so that unauthorized persons will not be able to access them. An encrypted link between your personal data and the answers collected in the survey ensures that the researchers will not be able to connect your answers to any identifiable information. The results of the project will be presented in research reports. The research reports will only contain summary statistics about the participants, that is, the number of participants, the proportion of women, age distribution, etc.

Linkage with Administrative Registers

With your survey participation, you help us to better understand decision-making around COVID-19 vaccination. To better understand COVID-19 vaccination decisions, the public health agency will need to link your survey responses to vaccination data. In order to link the data, you will therefore be asked for your social security number. **Your social security number will be collected at the very beginning on a one-page survey. After, you will be forwarded to a different survey so that nobody--neither the researchers, nor the survey provider, nor the Public Health Agency, nor any other person--will be able to link your social security number to your survey responses.** Your social security number will be kept completely confidential and the researchers working with your survey responses will not have access to it.

Click here to learn more details about the procedure to ensure confidentiality.

Pop-up: Enkätfabriken, the survey provider, will collect your social security number (and no other data) on the next screen of this survey. Enkätfabriken will then send the social security number to the public health agency along with an anonymized random number. Then the public health agency will match the anonymized random number with data on COVID-19 vaccinations using your social security number and afterwards delete your social security number. The public health agency will finally send the anonymized data consisting of the anonymized random number and the vaccination data to the researchers.

The researchers have only access to the answers to the second part of the survey (which does not include questions about your social security number or any other questions that would allow them to identify you). The dataset from second part of the survey includes the same anonymized random number the public health agency has. The researchers can therefore link the vaccination data that they receive from the public health agency with the answers to the second part of the survey using the anonymized random number. Note that the anonymized random number does not allow the researchers to identify you.

This procedure means that:

- **Neither the researchers, nor Enkätfabriken, nor the Public Health Agency, nor any other person will be able to link your social security number to your survey responses.**
- **Neither the researchers of the study nor any other third parties will ever have access to your social security number.**

Your anonymity will hence always be protected.

This matching between the survey data and vaccination data will happen twice: once in mid 2021 and once in late 2021-early 2022. Latest at the end of 2022, Enkätfabriken will delete all social security ID data.

How do I get information about the project results?

Research reports can be ordered from the responsible researcher (see below). It usually takes time (more than a year) before there is a complete report. Aggregate information about other participants' decisions can be obtained on request.

Responsible researcher

Lund University is responsible for the project. The researcher responsible is Professor Erik Wengström at the Department of Economics.

Erik.wengstrom@nek.lu.se

Criteria for participation

This study consists only of answering questions. However, you must not participate if any of the following are true: you have already received a COVID-19 vaccination, you are pregnant, you have previously experienced an allergic reaction that required hospital care, or you have ever experienced a severe allergic reaction after you got a vaccine. To assess whether it is appropriate for you to participate in the study, we must therefore first ask the following question.

Is any of the following statements true?
- I have already received a COVID-19 vaccination
- I am pregnant
- I have previously experienced an allergic reaction that required hospital care
- I have previously experienced a severe reaction after I got a vaccine

[] Yes, at least one of the statements is true
[] No, none of the statements is true

Your participation is voluntary and you can choose to cancel your participation at any time by pressing a button that cancels the questionnaire. If you choose not to participate or want to cancel your participation, you do not need to state why.

[] I have received information on data confidentiality and I agree with the linkage of my social security number to the COVID-19 vaccination register
[] I agree to participate in the study

Questionnaire items

Screen: Social security number

- **Please, type your social security number:**

Note:

- Remember that your social security number will be kept completely confidential and that nobody (neither the researchers, nor the survey provider, nor the Public Health Agency, nor any other person) will be able to link your social security number to your survey responses.
- Your response here will be saved by Enkätfabriken, the survey provider, and the responses in the following survey will be saved by the researchers, so neither Enkätfabriken nor the researchers will be able to link your survey responses to your social security number.
- Click **here** to learn more details about the procedure to ensure confidentiality.

When clicking Next Page, subjects are automatically transferred to another survey

Screen: Preferences and personality

- In the following, we will ask you several questions about your willingness to act in certain ways.

  Please indicate your answer on a scale from 0 to 10. A 0 means "completely unwilling to do so," and a 10 means "very willing to do so." You can also use any number between 0 and 10 to indicate where you fall on the scale, using 0, 1, 2, 3, 4, 5, 6, 7, 8, 9, or 10.

  - **How willing are you to give to good causes without expecting anything in return?**
  - **In general, how willing are you to take risks?**
  - **How willing are you to give up something that is beneficial for you today in order to benefit more from that in the future?**

  (Response scale: 0-10)

- How well do the following statements describe you as a person?

  Please indicate your answer on a scale from 0 to 10. A 0 means "does not describe me at all," and a 10 means "describes me perfectly." You can use any number between 0 and 10 to indicate where you fall on the scale, using 0, 1, 2, 3, 4, 5, 6, 7, 8, 9, or 10.

  - **When someone does me a favor, I am willing to return it.**
  - **I assume that people have only the best intentions.**
  - **I postpone starting on things I dislike to do.**
  - **It is important for me to always behave properly and to avoid doing anything people would say is wrong.**

  (Response scale: 0-10)

Screen: COVID-19 and vaccine knowledge

- We will now ask you some questions about COVID-19 and vaccinations.
  - **Have you ever tested positive for COVID-19 or COVID-19 antibodies?**
  - **Are you in an at-risk group for COVID-19?**
  - **Have you already been eligible for getting a COVID-19 vaccination?**

(Response scale: Yes/No/I don't know)

- To what extent do you agree with the following statements:
  - **In general, COVID-19 vaccines are safe.**
  - **Diseases like autism, multiple sclerosis, and diabetes might be triggered through vaccination.**
  - **I am worried about the side effects from COVID-19 vaccines.**
  - **I am afraid of the needles used for vaccination.**

(Response scale: Completely disagree, Disagree, Neither agree nor disagree, Agree, Completely agree)

Screen: Demographics

We will now ask you some questions about your personal circumstances.

- **What year were you born?**

(Response scale: 1955-2005)

- **Do you identify yourself as a woman or a man?**

(Response scale: woman, man, neither man nor woman)

- **What describes your civil status best?**

(Response scale: single, sarbo, couple, married, others)

- **How many children live in your household?**

(Response scale: no children, 1 child, 2 children, 3 children, 4 children, 5 or more children)

- **What is your employment status?**

(Response scale: work, unemployed, student, pensioner, others)

- **What education do you have (fill in the highest you have)?**

(Response scale: elementary or lower, high-school, professional training, ongoing university studies, university studies, research studies)

- **In which region do you live?**

(Response scale: Blekinge, Dalarna, Gotland, Gävleborg, Halland, Jämtland, Jönköping, Kalmar, Kronoberg, Norrbotten, Skåne, Stockholm, Södermanland, Uppsala, Värmland, Västerbotten, Västernorrland, Västmanland, Västra Götaland, Örebro, Östergötland)

- **Where was your father born?**

(Response scale: Sweden, Another European country, North America, South America, Africa, Middle-east, Rest of Asia, Oceania)

- **Where was your mother born?**

(Response scale: Sweden, Another European country, North America, South America, Africa, Middle-east, Rest of Asia, Oceania)

- **How much is your household's total income per month after taxes including public benefits? Calculate also your loan if you are a student. Please answer even if you are not sure.**

(Response scale: 0-5000kr, 5001-10000kr, 10001-15000kr, 15001-20000kr, 20001-25000kr, 25001-30000kr, 30001-35000kr, 35001-40000kr , 40001-45000kr, 45001-50000kr, 50000kr-55000kr, more than 55000kr)

Interventions

1. Control condition

We would like to encourage you to get a COVID-19 vaccine as soon as possible, ideally within the first 30 days after the vaccine becomes available to you.

Reminder

Thank you again for participating in the survey on COVID-19 vaccination.

We would like to encourage you to get a COVID-19 vaccine as soon as possible, ideally within the first 30 days after the vaccine becomes available to you.

Click here to get information about how you can **book an appointment for vaccination against COVID-19 in your region.**

Erik Wengström, Professor at Lund University

2. Incentives condition

We would like to encourage you to get a COVID-19 vaccine as soon as possible, ideally within the first 30 days after the vaccine becomes available to you.

We offer you SEK 200 if you get a first shot of a COVID-19 vaccine within the first 30 days after the vaccination becomes available to you*.

* available means that vaccination started for people in your age category and region. If the vaccine has already become available to you, you will receive the SEK 200 if you get vaccinated within 30 days. We will pay you as soon as possible, but latest before the end of the year.

We will check with the public health agency whether you vaccinated within 30 days the vaccine has become available to you. If you got vaccinated, we will send you a gift card with a value of SEK 200.[1] As explained previously, your data will be treated confidentially.

The following picture is a certificate that ensures that you will be paid SEK 200 if you get a COVID-19 vaccine within 30 days after it becomes available to you. Please take a picture and/or save this document.

> *I, Dr. Erik Wengström, Professor at Lund University and co-researcher of this study, guarantee that you will receive a SEK 200 voucher via e-mail if you take a first shot of a COVID-19 vaccine within 30 days after people in your region and age group can take the vaccine.*
>
> 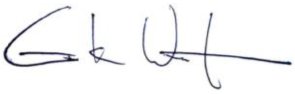
>
> *Erik Wengström*
>
> *Professor at Lund University*

- **I understand that if I take a first shot of a COVID-19 vaccine within one month after which it became available to me, I will be paid $10**
  (Response scale: Yes/No)

Reminder

Thank you again for participating in the survey on COVID-19 vaccination.

We would like to encourage you to get a COVID-19 vaccine as soon as possible, ideally within the first 30 days after the vaccine becomes available to you.

Remember that we will pay you SEK 200 if you get a first shot of a COVID-19 vaccine within one month after which it became available to you.

Click here to get information about how you can **book an appointment for vaccination against COVID-19 in your region.**

Erik Wengström, Professor at Lund University

[1] This card is a SuperPresentKort that gives you choices among all present cards at gogift.com – you can choose from more than 150 retail stores, including Zalando, Åhlens, H&M, Pressbyron, McDonalds, Jack&Jones, and many more.

3. Social impact condition

We would like to encourage you to get a COVID-19 vaccine as soon as possible, ideally within the first 30 days after the vaccine becomes available to you.

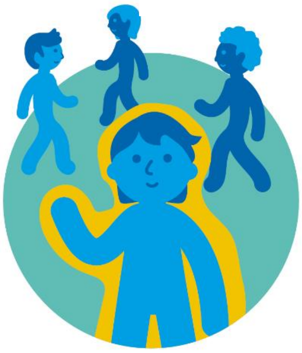

The COVID-19 vaccine not only protects you, but also protects people around you (e.g., family, friends, neighbors, work colleagues, and local shopkeepers). Now, we would like you to make a list of the 4 people who will benefit if you get the vaccine.

Please write down their first name (if you know the person's name) and how they are related to you. Note that none of this information will be matched with your personal data.

As an example, please take a look at the list of Erik Wengström, co-researcher of this study:

Ann, Mother
Anders, Friend in a risk group
Mohammed, My local pizza cook
Johan and his schoolmates, Students in the local high school

Please write down your own list with peoples' first name and how they are related to you in the following textboxes: (Response in 4 textboxes)

Reminder

Thank you again for participating in the survey on COVID-19 vaccination.

We would like to encourage you to get a COVID-19 vaccine as soon as possible, ideally within the first month after the vaccine becomes available to you.

We would like to remind you that the COVID-19 vaccine not only protects you, but also protects the people around you (e.g., family, neighbors, work colleagues, and local shopkeepers). When you participated in our survey, you made a list consisting of four people that would benefit if you took the vaccine. We would like that you think about these four people when you consider taking the vaccine.

Click here to get information about how you can **book an appointment for vaccination against COVID-19 in your region.**

Erik Wengström, Professor at Lund University

4. Argument condition

We would like to encourage you to get a COVID-19 vaccine as soon as possible, ideally within the first 30 days after the vaccine becomes available to you.

There are people who do not plan to get the vaccine soon after it becomes available for them. We would like to ask you to write down arguments that you think could best convince another person to change his/her mind and get the vaccine as soon as possible, ideally within 30 days after it becomes available to him/her.

We will also give you the opportunity to share your argument with a person that does not plan to get the vaccine. We will randomly select the arguments of ten participants in this study and control that the participant agreed to share the argument. If the participant agreed, we will then share the argument with a person that does NOT plan to get the vaccine and does NOT have an increased risk of side effects from COVID-19 vaccination. You remain anonymous and your argument will never be connected to your personal data.

Note: You can write down an argument directly. lternatively, you are also welcome to look for arguments, for example on the the Swedish health authority website.

Please write your arguments in the following textbox:

(Response scale: textbox)

[] I agree that my arguments may be shared anonymously with someone who does NOT plan to get the vaccine and does NOT have an increased risk of side effects from COVID-19 vaccination.

[] I do not want that my arguments are shared.

Reminder

Thank you again for participating in the survey on COVID-19 vaccination.

We would like to encourage you to get a COVID-19 vaccine as soon as possible, ideally within the first 30 days after the vaccine becomes available to you.

We would like to remind you that you wrong an argument to convince another person to take the vaccine as soon as possible, ideally within the first 30 days after it becomes available to them. We would like you to think about an argument that you think could best convince another person to get the vaccine.

Click here to get information about how you can **book an appointment for vaccination against COVID-19 in your region.**

Erik Wengström, Professor at Lund University

5. Information Treatment

Screen 1

We would like to encourage you to get a COVID-19 vaccine as soon as possible, ideally within the first 30 days after the vaccine becomes available to you.

In the following, we would like to ask you to fill out a short quiz about the effectiveness and safety of the COVID-19 vaccine.

Screen 2

Quiz Question 1: Effectiveness of COVID-19 vaccines

What do you think, how effective are the COVID-19 vaccines that are used in Sweden for your age group in reducing deaths from COVID-19?

(Response scale: 0 – 100%)

Screen 3

The information below is confirmed by the virologist Prof. Niklas Arnberg at Umeå University:

Trials have shown that **the currently approved vaccines in Sweden strongly protect against the virus**. In fact, results from millions of vaccinated people in Israel show that the Pfizer-BioNTech **vaccine prevents 97% of deaths from COVID-19.**

Before you move to the next screen, please now provide the correct answer to quiz question 1:

How effective are COVID-19 vaccines in reducing deaths from COVID-19?

(Response scale: 0 – 100%)

Screen 4

Quiz Question 2: Safety of the vaccine

What do you think, how many deaths in the EU and the USA have been confirmed to have a connection to the COVID-19 vaccines that are used in Sweden for your age group?

(Response scale: 0 deaths, 1-5 deaths, 5-10 deaths, 11-20 deaths, 21-50 deaths, 51-100 deaths, 101-200 deaths, 201-500 deaths, 501-1’000 deaths, 1’001-2’000 deaths, 2’001-5’000 deaths, 5’001-10’000 deaths, >10’000 deaths)

Screen 5

The information below is confirmed by the virologist Prof. Niklas Arnberg at Umeå University:

The vaccines have shown to be safe.

Serious side-effects are very rare. The vaccines that are used in Sweden for your age group can rarely create side effects in the form of allergic reactions. Studies indicate that fewer than 3 people per 10,000 vaccinated get such an effect. Most of them have previously had allergies, and everyone in the studies recovered himself/herself completely.

After more than half of USAs adult population has vaccinated

The vaccines only very rarely trigger side-effects in the form of allergic (anaphylactic) reactions in 5 to 10 people out of 1,000,000 vaccinated people or in 0.001% of vaccinated people. Most people who developed these reactions have had a history of allergies, and all of them have fully recovered.

After more than half of the US adult population has been vaccinated, no causal link has been established between deaths and the vaccines used in Sweden for your age group. Within the EU, over 120 million doses of the Pfizer-BioNTech vaccine have been administered. Here, too, no link has been established between deaths and the vaccine.

Please now provide the correct answer to the quiz question:

How many deaths in the EU and the USA have been confirmed to have a connection to the COVID-19 vaccines that are used in Sweden for your age group?

(Response scale: 0 deaths, 1-5 deaths, 5-10 deaths, 11-20 deaths, 21-50 deaths, 51-100 deaths, 101-200 deaths, 201-500 deaths, 501-1’000 deaths, 1’001-2’000 deaths, 2’001-5’000 deaths, 5’001-10’000 deaths, >10’000 deaths)

Reminder

Thank you again for participating in the survey on COVID-19 vaccination.

We would like to encourage you to get a COVID-19 vaccine as soon as possible, ideally within the first 30 days after the vaccine becomes available to you.

In the study, we asked two questions about the efficacy and safety of the COVID-19 vaccines. Based on the studies we referred to, the following applies:

- **The vaccines are very effective. The COVID-19 vaccines used in Sweden for your age group have been shown to reduce COVID-19 deaths by 97%.**
- **The vaccines have been shown to be safe. After more than 300 million vaccinated people in the EU and the US, no link has been established between deaths and the vaccines used for your age group.**

Click here to get information about how you can **book an appointment for vaccination against COVID-19 in your region.**

Erik Wengström, Professor at Lund University

Intentions

Screen: Vaccine intention 1 (main outcome for intentions)

- **Do you think you will get a first shot of a COVID-19 vaccine within the first 30 days after the vaccine becomes available to you?***
  * Available means that vaccinations started for people in your age group in your region
  (Response scale: No/Yes)

Screen: Vaccination Intention 2

- **We understand that there is always some uncertainty regarding all decisions. From 0% to 100%, what do you think are the chances that you will choose to get a first shot of a COVID-19 vaccine within the first 30 days after the vaccine becomes available to you?**

  (Response scale: 0-100)

Screen: Vaccination Intention 3

- **When do you think you will get a COVID-19 vaccine after the vaccine becomes available to you?**

  (Response scale: within 1 week, within 2 weeks, within 3 weeks, within 1 month, within 2 months, within 3 months, within 6 months, within 12 months, after 12 months, never)

End of study

[Brackets indicate for which treatment each sentence was displayed]

We would like to encourage you to get a COVID-19 vaccine as soon as possible, ideally within the first month after the vaccine becomes available to you. [*Control condition, incentives condition, social impact condition, argument condition, information condition*]

- Remember that we will pay you SEK 200 if you get a first shot of a COVID-19 vaccine within 30 days after it becomes available to you. *[Incentives condition]*
- Remember that the COVID-19 vaccine not only protects you, but also protects the people around you (e.g., family, neighbors, work colleagues, and local shopkeepers). You made the following list of 4 people who will benefit if you get the vaccine:
  [List of the four people listed by the participant]
  [Social impact condition]
- Remember, that you wrote the following argument to convince another person to get the vaccine as soon as possible, ideally within 30 days after it is available to him/her:
  [Argument]
  [*Argument condition*]
- Remember that the COVID-19 vaccines that are used in Sweden for your age group are both effective and safe. Studies show that the Pfizer-BioNTech vaccine prevents 97% of the deaths from COVID-19. After hundreds of millions have vaccinated there has not been found any connection between deaths and the vaccines. [*Information condition]*

Click here to get information about how you can **book an appointment for vaccination against COVID-19 in your region.** [*Control condition, incentives condition, social impact condition, argument condition, information condition*]

Note, do not forget to submit your answers even if you click on the link above! [*Control condition, incentives condition, social impact condition, argument condition, information condition*]

Thank you for participating in our study!

If you have any questions about the study, contact the responsible researcher Professor Erik Wengström, Department of Economics at Lund University.

Address: Department of Economics, Box 7080, 220 07 Lund.

Email: erik.wengstrom@nek.lu.se.

Phone: 046 222 0123.

Click on the arrow at the bottom to submit your answers.

References and Notes

1. B. S. Graham, Rapid COVID-19 vaccine development. *Science* **368**, 945–946 (2020). doi:10.1126/science.abb8923 Medline

2. D. Skegg, P. Gluckman, G. Boulton, H. Hackmann, S. S. A. Karim, P. Piot, C. Woopen, Future scenarios for the COVID-19 pandemic. *Lancet* **397**, 777–778 (2021). doi:10.1016/S0140-6736(21)00424-4 Medline

3. E. Callaway, Delta coronavirus variant: Scientists brace for impact. *Nature* **595**, 17–18 (2021). doi:10.1038/d41586-021-01696-3 Medline

4. K. L. Milkman, J. Beshears, J. J. Choi, D. Laibson, B. C. Madrian, Using implementation intentions prompts to enhance influenza vaccination rates. *Proc. Natl. Acad. Sci. U.S.A.* **108**, 10415–10420 (2011). doi:10.1073/pnas.1103170108 Medline

5. T. Rogers, K. L. Milkman, L. K. John, M. I. Norton, Beyond good intentions: Prompting people to make plans improves follow-through on important tasks. *Behav. Sci. Policy* **1**, 33–41 (2015). doi:10.1353/bsp.2015.0011

6. K. M. Harris, J. Maurer, A. L. Kellermann, Influenza vaccine—Safe, effective, and mistrusted. *N. Engl. J. Med.* **363**, 2183–2185 (2010). doi:10.1056/NEJMp1012333 Medline

7. A. Oza, Studies probe how payouts affect U.S. vaccination rates. *Science* **373**, 611 (2021). doi:10.1126/science.373.6555.611 Medline

8. K. Terrell, "These Companies Are Paying Employees to Get Vaccinated" (2021); www.aarp.org/work/working-at-50-plus/info-2021/companies-paying-employees-covid-vaccine.html.

9. Reuters, "Biden wants state, local govts to pay $100 to newly vaccinated Americans" (2021); www.reuters.com/world/us/biden-wants-state-local-govts-give-100-newly-vaccinated-americans-treasury-2021-07-29/.

10. J. Haushofer, C. J. E. Metcalf, Which interventions work best in a pandemic? *Science* **368**, 1063–1065 (2020). doi:10.1126/science.abb6144 Medline

11. G. Charness, U. Gneezy, Incentives to exercise. *Econometrica* **77**, 909–931 (2009). doi:10.3982/ECTA7416

12. K. G. Volpp, L. K. John, A. B. Troxel, L. Norton, J. Fassbender, G. Loewenstein, Financial incentive-based approaches for weight loss: A randomized trial. *JAMA* **300**, 2631–2637 (2008). doi:10.1001/jama.2008.804 Medline

13. K. G. Volpp, A. B. Troxel, M. V. Pauly, H. A. Glick, A. Puig, D. A. Asch, R. Galvin, J. Zhu, F. Wan, J. DeGuzman, E. Corbett, J. Weiner, J. Audrain-McGovern, A randomized, controlled trial of financial incentives for smoking cessation. *N. Engl. J. Med.* **360**, 699–709 (2009). doi:10.1056/NEJMsa0806819 Medline

14. A. V. Banerjee, E. Duflo, R. Glennerster, D. Kothari, Improving immunisation coverage in rural India: Clustered randomised controlled evaluation of immunisation campaigns with and without incentives. *BMJ* **340**, c2220 (2010). doi:10.1136/bmj.c2220 Medline

15. N. Lacetera, M. Macis, R. Slonim, Economic rewards to motivate blood donations. *Science* **340**, 927–928 (2013). doi:10.1126/science.1232280 Medline

16. E. L. Deci, Effects of externally mediated rewards on intrinsic motivation. *J. Pers. Soc. Psychol.* **18**, 105–115 (1971). doi:10.1037/h0030644

17. U. Gneezy, A. Rustichini, Pay enough or don't pay at all. *Q. J. Econ.* **115**, 791–810 (2000). doi:10.1162/003355300554917

18. S. Bowles, Policies designed for self-interested citizens may undermine "the moral sentiments": Evidence from economic experiments. *Science* **320**, 1605–1609 (2008). doi:10.1126/science.1152110 Medline

19. C. Mellström, M. Johannesson, Crowding out in blood donation: Was Titmuss right? *J. Eur. Econ. Assoc.* **6**, 845–863 (2008). doi:10.1162/JEEA.2008.6.4.845

20. U. Gneezy, S. Meier, P. Rey-Biel, When and why incentives (don't) work to modify behavior. *J. Econ. Perspect.* **25**, 191–210 (2011). doi:10.1257/jep.25.4.191

21. E. A. Largent, F. G. Miller, Problems with paying people to be vaccinated against COVID-19. *JAMA* **325**, 534–535 (2021). doi:10.1001/jama.2020.27121 Medline

22. K. G. Volpp, G. Loewenstein, A. M. Buttenheim, Behaviorally informed strategies for a national COVID-19 vaccine promotion program. *JAMA* **325**, 125–126 (2021). Medline

23. J. Savulescu, Good reasons to vaccinate: Mandatory or payment for risk? *J. Med. Ethics* **47**, 78–85 (2021). doi:10.1136/medethics-2020-106821 Medline

24. N. S. Jecker, What money can't buy: An argument against paying people to get vaccinated. *J. Med. Ethics* 10.1136/medethics-2021-107235 (2021). doi:10.1136/medethics-2021-107235 Medline

25. R. Thaler, C. Sunstein, *Nudge: Improving Decisions About Health, Wealth, and Happiness* (Penguin Books, 2008).

26. M. S. Patel, K. G. Volpp, D. A. Asch, Nudge units to improve the delivery of health care. *N. Engl. J. Med.* **378**, 214–216 (2018). doi:10.1056/NEJMp1712984 Medline

27. K. L. Milkman, M. S. Patel, L. Gandhi, H. N. Graci, D. M. Gromet, H. Ho, J. S. Kay, T. W. Lee, M. Akinola, J. Beshears, J. E. Bogard, A. Buttenheim, C. F. Chabris, G. B. Chapman, J. J. Choi, H. Dai, C. R. Fox, A. Goren, M. D. Hilchey, J. Hmurovic, L. K. John, D. Karlan, M. Kim, D. Laibson, C. Lamberton, B. C. Madrian, M. N. Meyer, M. Modanu, J. Nam, T. Rogers, R. Rondina, S. Saccardo, M. Shermohammed, D. Soman, J. Sparks, C. Warren, M. Weber, R. Berman, C. N. Evans, C. K. Snider, E. Tsukayama, C. Van den Bulte, K. G. Volpp, A. L. Duckworth, A megastudy of text-based nudges encouraging patients to get vaccinated at an upcoming doctor's appointment. *Proc. Natl. Acad. Sci. U.S.A.* **118**, e2101165118 (2021). doi:10.1073/pnas.2101165118 Medline

28. S. DellaVigna, E. Linos, "RCTs to scale: Comprehensive evidence from two nudge units" (Working Paper no. 27594, National Bureau of Economic Research, 2021); www.nber.org/papers/w27594.

29. H. Dai, S. Saccardo, M. A. Han, L. Roh, N. Raja, S. Vangala, H. Modi, S. Pandya, M. Sloyan, D. M. Croymans, Behavioural nudges increase COVID-19 vaccinations. *Nature* **597**, 404–409 (2021). doi:10.1038/s41586-021-03843-2 Medline

30. R. Thaler, "More than nudges are needed to end the pandemic," *New York Times* (2021); www.nytimes.com/2021/08/05/business/vaccine-pandemic-nudge-passport.html.

31. C. Jarrett, R. Wilson, M. O'Leary, E. Eckersberger, H. J. Larson; SAGE Working Group on Vaccine Hesitancy, Strategies for addressing vaccine hesitancy - A systematic review. *Vaccine* **33**, 4180–4190 (2015). doi:10.1016/j.vaccine.2015.04.040 Medline

32. G. B. Chapman, M. Li, H. Colby, H. Yoon, Opting in vs opting out of influenza vaccination. *JAMA* **304**, 43–44 (2010). doi:10.1001/jama.2010.892 Medline

33. L. Korn, R. Böhm, N. W. Meier, C. Betsch, Vaccination as a social contract. *Proc. Natl. Acad. Sci. U.S.A.* **117**, 14890–14899 (2020). doi:10.1073/pnas.1919666117 Medline

34. P. Campos-Mercade, A. N. Meier, F. H. Schneider, E. Wengström, Prosociality predicts health behaviors during the COVID-19 pandemic. *J. Public Econ.* **195**, 104367 (2021). doi:10.1016/j.jpubeco.2021.104367 Medline

35. P. Campos-Mercade, A. Meier, F. H. Schneider, S. Meier, D. Pope, E. Wengström, Data and code for "Monetary incentives increase COVID-19 vaccinations," Version 1, Zenodo (2021); https://doi.org/10.5281/zenodo.5529625.

36. P. Campos-Mercade, A. Meier, F. Schneider, S. Meier, D. Pope, E. Wengström, "Behavioral interventions, economic preferences and vaccination uptake," RCT ID AEARCTR-0007652, AEA RCT Registry (2021); https://doi.org/10.1257/rct.7652-2.0.

37. A. Falk, A. Becker, T. Dohmen, B. Enke, D. Huffman, U. Sunde, Global evidence on economic preferences. *Q. J. Econ.* **133**, 1645–1692 (2018). doi:10.1093/qje/qjy013

38. B. W. Tuckman, The development and concurrent validity of the procrastination scale. *Educ. Psychol. Meas.* **51**, 473–480 (1991). doi:10.1177/0013164491512022

39. C. R. Wells, A. Huppert, M. C. Fitzpatrick, A. Pandey, B. Velan, B. H. Singer, C. T. Bauch, A. P. Galvani, Prosocial polio vaccination in Israel. *Proc. Natl. Acad. Sci. U.S.A.* **117**, 13138–13144 (2020). doi:10.1073/pnas.1922746117 Medline

40. E. J. Haas, F. J. Angulo, J. M. McLaughlin, E. Anis, S. R. Singer, F. Khan, N. Brooks, M. Smaja, G. Mircus, K. Pan, J. Southern, D. L. Swerdlow, L. Jodar, Y. Levy, S. Alroy-Preis, Impact and effectiveness of mRNA BNT162b2 vaccine against SARS-CoV-2 infections and COVID-19 cases, hospitalisations, and deaths following a nationwide vaccination campaign in Israel: An observational study using national surveillance data. *Lancet* **397**, 1819–1829 (2021). doi:10.1016/S0140-6736(21)00947-8 Medline

41. S. Loomba, A. de Figueiredo, S. J. Piatek, K. de Graaf, H. J. Larson, Measuring the impact of COVID-19 vaccine misinformation on vaccination intent in the UK and USA. *Nat. Hum. Behav.* **5**, 337–348 (2021). doi:10.1038/s41562-021-01056-1 Medline

42. Trafikverket, "Analysmetod och samhällsekonomiska kalkylvärden för transportsektorn," ASEK 7.0 (2020); www.trafikverket.se/for-dig-i-branschen/Planera-och-utreda/Planerings--och-analysmetoder/Samhallsekonomisk-analys-och-trafikanalys/asek-analysmetod-och-samhallsekonomiska-kalkylvarden/.

43. S. Gupta, J. Cantor, K. I. Simon, A. I. Bento, C. Wing, C. M. Whaley, Vaccinations against COVID-19 may have averted up to 140,000 deaths in the United States. *Health Aff.* **40**, 1465–1472 (2021). doi:10.1377/hlthaff.2021.00619 Medline

44. A. Galvani, S. M. Moghadas, E. C. Schneider, "Deaths and hospitalizations averted by rapid U.S. vaccination rollout" (Commonwealth Fund, 2021); https://doi.org/10.26099/wm2j-mz32.
